# TREM-1-Associated Neutrophil Extracellular Trap Formation is Linked to IVIG Resistance in Kawasaki Disease: A Convergent Transcriptomic and Prospective Validation Study

**DOI:** 10.1007/s10875-026-02025-x

**Published:** 2026-04-21

**Authors:** Sibao Wang, Gang Luo, Zhixian Ji, Silin Pan

**Affiliations:** Heart Center, Women and Children’s Hospital, Qingdao University, Qingdao, 266034 China

**Keywords:** Machine Learning, Prediction Model, TREM-1, IVIG Resistance, Kawasaki Disease, Neutrophil Extracellular Traps

## Abstract

Kawasaki disease (KD) remains the leading cause of acquired heart disease in children. While intravenous immunoglobulin (IVIG) represents standard therapy, approximately 10–20% of patients exhibit treatment refractoriness associated with significantly elevated coronary artery lesion risk. Current risk stratification relies on clinical parameters—fever duration or nonspecific inflammatory markers—which fail to capture distinct immunopathological features associated with treatment failure. To bridge this gap, we employed a convergent strategy integrating transcriptomic discovery from two independent cohorts with prospective protein-level and cellular validation in a clinical cohort. Three machine learning models—LASSO regression, Random Forest, and XGBoost—independently identified TREM1 (Triggering Receptor Expressed on Myeloid cells-1) as a core resistance predictor. Prospective validation in 117 KD patients demonstrated that plasma soluble TREM-1 (sTREM-1) was markedly elevated in IVIG-resistant cases (median 1247 vs. 856 pg/mL, *P* < 0.001) and effectively differentiated KD from other febrile illnesses. Mechanistically, sTREM-1 elevation showed a moderate positive correlation with myeloperoxidase-DNA complexes (ρ = 0.612, *P* < 0.001), indicating that TREM-1-associated neutrophil hyperactivation is associated with excessive neutrophil extracellular trap (NET) formation and vascular injury. External validation (GSE63881) confirmed TREM1 upregulation (2.1-fold) in resistant patients. Incorporating sTREM-1 into a multivariable prediction model achieved area under curve 0.884 using Firth’s penalized logistic regression (optimism-corrected AUC 0.871) in this derivation cohort, significantly outperforming traditional clinical scores. These findings suggest TREM-1-associated NETosis as a potential contributor to the pathobiology of IVIG resistance. As a TRIPOD Type 1b derivation study, external clinical validation in independent multi-center cohorts is required prior to clinical implementation.

**Supplementary Information:**

The online version contains supplementary material available at 10.1007/s10875-026-02025-x.

## Introduction

Among pediatric cardiac conditions of acquired etiology, Kawasaki disease (KD) holds an unfortunate distinction as a leading cause of long-term cardiovascular morbidity. The introduction of high-dose intravenous immunoglobulin (IVIG) has significantly altered the therapeutic landscape, decreasing the incidence of coronary artery lesions (CAL) from 25% to around 4% [1]. This success, however, is mitigated by a substantial clinical challenge: around 10–20% of affected children are resistant to initial immunotherapy, encountering a two- to five-fold increase in the risk of coronary abnormalities [1, 2]. Early identification and aggressive management of these high-risk patients represents a critical priority, yet predicting treatment failure remains frustratingly elusive.

Traditional risk-scoring systems, such as the Kobayashi score, function adequately within their Japanese derivation populations but consistently lack sufficient sensitivity in heterogeneous cohorts, frequently resulting in prediction accuracies marginally superior to chance [3, 4]. This limitation likely arises from a fundamental conceptual deficiency: these models depend on clinical phenotypes—fever duration, routine laboratory values—which function merely as downstream proxies for systemic inflammation rather than representing the unique immunopathological factors contributing to treatment failure. The field urgently requires biomarkers grounded in disease biology rather than phenotypic surrogates of systemic inflammation.

Substantial evidence implicates dysregulated innate immunity, especially neutrophil hyperactivation, as a central pathogenic driver of KD. Recent single-cell studies have documented the expansion of CD177-positive neutrophil populations displaying hyperactivated transcriptional signatures in affected children [5]. Neutrophil extracellular traps (NETs), which are web-like DNA structures released by activated neutrophils, build up during the acute phase. They can cause direct damage to the vascular endothelium through oxidative stress and inflammasome activation [6–9]. The critical question remains: what upstream molecular “switch” initiates and sustains this excessive neutrophil activation specifically in IVIG-resistant patients?

We hypothesized that a key regulator exists at the apex of this inflammatory cascade, serving both as a robust predictive biomarker and a specific therapeutic target. Instead of using single-dataset analyses that are prone to noise and batch effects, we used a convergent transcriptomic strategy. We aimed to find a consensus gene signature that could predict resistance with as few false positives as possible by combining transcriptomic profiles from two separate discovery cohorts and using three different machine learning algorithms. This computational finding was subsequently corroborated in a prospective clinical cohort, wherein we evaluated the candidate biomarker’s efficacy in forecasting treatment outcomes and its mechanistic function in the NETosis and vascular injury pathway.

## Methods

### Study Design and Participant Cohorts

To guarantee the identification of robust, generalizable biomarkers, we utilized a two-stage design that integrated computational discovery with prospective clinical validation. During the discovery phase, we created a large-scale meta-cohort by combining transcriptomic profiles from two separate datasets, GSE73461 [10] and GSE68004 [11], both of which came from the Gene Expression Omnibus. To prevent data leakage and make sure that external validation was strict, the third dataset (GSE63881 [12]) was only used for independent biological confirmation and not for the first feature selection. The clinical validation phase took place prospectively at Qingdao Women and Children’s Hospital. From January 2022 to December 2024, we enrolled 117 children diagnosed with KD according to American Heart Association criteria [1], alongside 18 febrile controls frequency-matched for age (within ± 6 months) and sex (fever > 38.5 °C for ≥ 3 days, confirmed non-KD etiology; Supplementary Table S1) and 19 healthy donors. Blood samples were taken within 48 h of admission during the acute febrile phase, which is the same time frame as the pre-treatment samples for patients with the target disease. To focus on the primary immune response, biological samples were obtained prior to IVIG administration. We did not include patients who had already received immunomodulatory treatment, had congenital heart defects, or had primary immunodeficiency. The study protocol complied with the Declaration of Helsinki and was sanctioned by the Institutional Ethics Committee (Reference: QDFYKY-2021-12), with written informed consent secured from all guardians.

## Clinical Definitions

IVIG resistance was characterized by persistent or recrudescent fever (axillary temperature ≥ 37.5 °C) recorded at least twice, with a minimum interval of 4 h, occurring 36–48 h following the completion of the initial IVIG infusion (2 g/kg administered over 10–12 h), in accordance with AHA 2017 guidelines [1]. Fever recrudescence was characterized as the reoccurrence of fever following an initial defervescence lasting 24 h or more. Echocardiography identified coronary artery lesions (CALs) within 72 h of diagnosis, characterized by a coronary artery internal diameter Z-score ≥ 2.5, computed using the Dallaire reference equations for the left main coronary artery and proximal right coronary artery segments [1].

## Transcriptomic Processing and Consensus Machine Learning

Raw microarray data underwent robust multi-array average (RMA) normalization [13] followed by ComBat batch effect correction to eliminate technical artifacts while preserving biological variance [14]. Differential expression analysis was performed using the limma package (adjusted *P* < 0.05, |log₂ fold-change|>0.5) [15].

To identify a highly stable predictive signature, we implemented a consensus machine learning framework utilizing three algorithmically distinct approaches: (1) LASSO regression (regularization parameter λ optimized via 10-fold cross-validation) [16]; (2) Random Forest classification (ranking feature importance via Mean Decrease Gini across 500 decision trees) [17]; and (3) XGBoost (gradient boosting with hyperparameter tuning via 5-fold cross-validation) [18]. Supplementary Table S5 shows the best hyperparameters for all three algorithms. This methodological choice merits emphasis: we only used these algorithms on the discovery cohorts (GSE73461 and GSE68004), and we kept the validation cohort (GSE63881) completely separate from the whole feature selection process. Strict temporal separation of GSE63881 from all feature selection steps ensured that data leakage, which would inflate performance estimates, was precluded. Only genes that all three independent algorithms agreed were in the top 20 were chosen as high-confidence candidate biomarkers. This approach was intentionally designed to minimize false discovery rates inherent to single-algorithm analyses. All transcriptomic analyses were performed in R (v4.3.1) using the following packages: limma (v3.56.2) for differential expression, sva (v3.48.0) for batch correction, glmnet (v4.1–8) for LASSO, randomForest (v4.7-1.1.1.1) for Random Forest, xgboost (v1.7.6.1) and SHAPforxgboost (v0.1.3) for XGBoost, and rms (v6.7-0) for calibration assessment. Network visualization was performed in Cytoscape (v3.10.1).

## Biomarker Quantification and Flow Cytometry

Blood samples (5 mL) were collected into EDTA anticoagulant tubes within 24 h of KD diagnosis and strictly prior to IVIG administration. Plasma was separated within 2 h of collection by centrifugation (2000×g, 15 min, 4 °C), aliquoted into 200 µL portions, and stored at − 80 °C. Samples experienced one freeze-thaw cycle prior to analysis. Laboratory personnel performing ELISA and flow cytometry were blinded to all clinical outcomes, including IVIG response status and coronary artery findings. Using commercial ELISA kits (JONLNBIO, China) and following the manufacturer’s instructions, we measured the levels of plasma soluble TREM-1 (sTREM-1) and S100A12. Myeloperoxidase-DNA (MPO-DNA) complexes, a well-established surrogate for in vivo NET formation, were measured using capture ELISA kit (Lcskit, ED-102148, China). Citrullinated histone H3 (CitH3) was detected by immunofluorescence using rabbit anti-CitH3 antibody (Abcam, ab5103) with confocal microscopy (Zeiss LSM 880). For ex vivo functional experiments, KD patient neutrophils (*n* = 12, pre-IVIG) were treated with LP17 (TREM-1 inhibitory peptide, 100 nM; GenScript) or scrambled control peptide for 4 h, and NET formation was quantified by MPO-DNA ELISA and CitH3 immunofluorescence. Healthy donor neutrophils (*n* = 8) were stimulated with rsTREM-1 (R&D Systems, #1278-TR; 0–100 ng/mL) with or without LP17 co-treatment (100 nM). All ELISA measurements were performed in duplicate (intra-assay CVs: 4.8%, 5.2%, and 6.1% for sTREM-1, S100A12, and MPO-DNA, respectively; inter-assay CVs: 7.3%, 8.1%, and 8.7%, respectively).

For cellular phenotyping, human neutrophils were isolated using a Human Neutrophil Isolation Kit (Solarbio, Cat. No. P8620, China). Neutrophil purity (> 95%) was confirmed by CD66b⁺gating via flow cytometry. Cells were stained with PE-conjugated anti-TREM-1 and APC-conjugated anti-CD66b antibodies (Proteintech). Surface expression was analyzed on a CytoFLEX S flow cytometer (Beckman Coulter), with data processed using FlowJo software (v10.8).

### Statistical Analysis

Continuous variables were assessed for normality using Shapiro-Wilk test. Normally distributed continuous variables were compared using Student’s t-test or one-way ANOVA; non-normally distributed variables were compared using Mann-Whitney U test or Kruskal-Wallis test with Dunn’s post-hoc correction.

Predictive models were constructed using multivariable logistic regression. Given the limited events-per-variable ratio (EPV = 9.3), we restricted the final model to a maximum of three predictors and applied Firth’s penalized logistic regression to reduce small-sample bias [19, 20]. Optimism-corrected performance was estimated via bootstrap internal validation (1,000 iterations) with uniform shrinkage factors applied to regression coefficients [21]. Full details of EPV considerations and sensitivity analyses are provided in Supplementary Methods.

The area under the receiver operating characteristic curve (AUC) was used to measure model discrimination, and the DeLong test was used to compare the results [22]. We used net reclassification improvement (NRI) and integrated discrimination improvement (IDI) [23] to see how much more predictive value the new biomarker added. Decision Curve Analysis (DCA) was performed to evaluate clinical utility [24] to see how useful the clinical was. All analyses were performed in R (v4.3.1), with a two-sided P-value of < 0.05 deemed significant.

## Results

### Machine Learning Identifies TREM-1 as a Convergent Biomarker Candidate

**Fig. 1:**
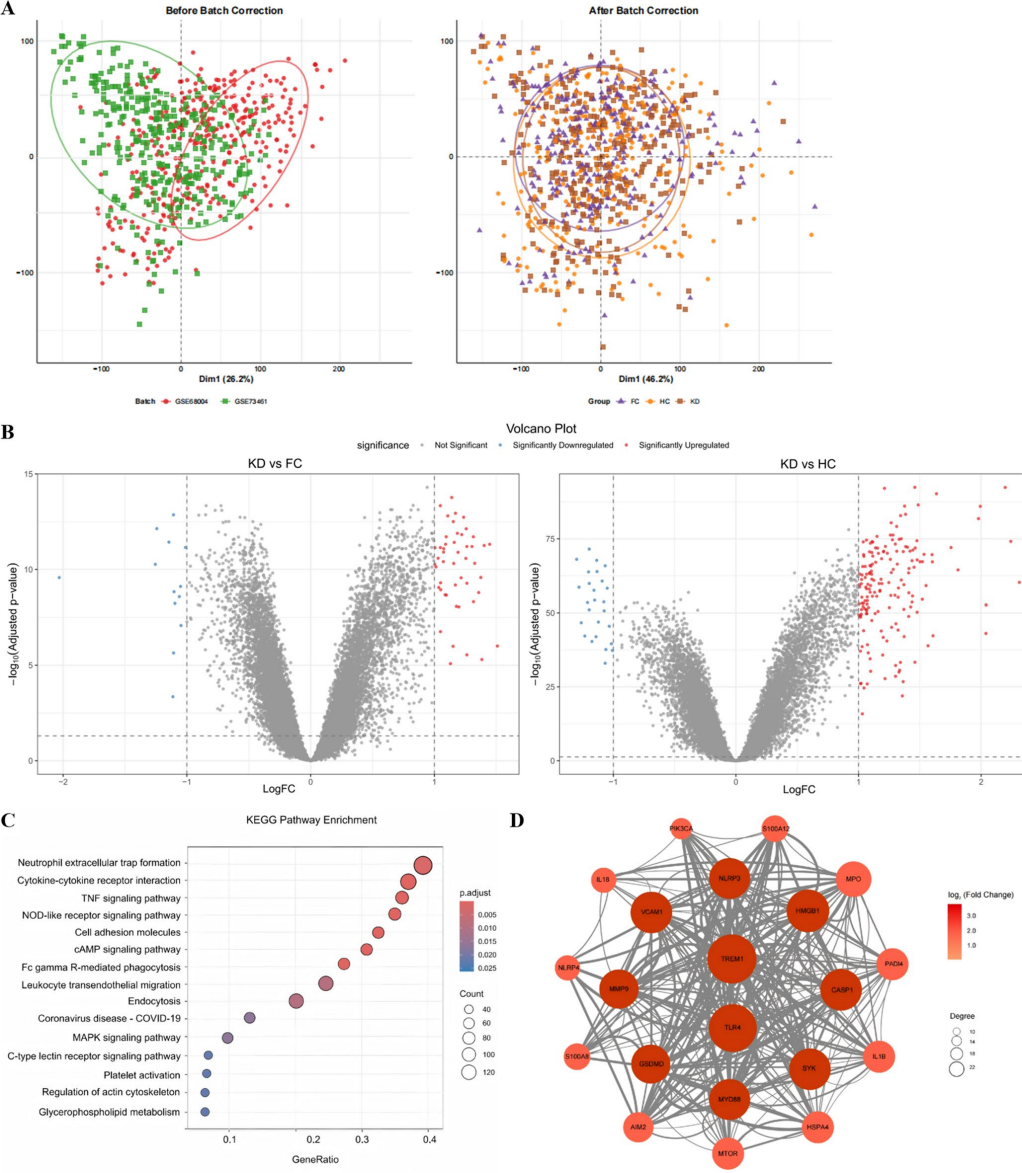
Integrated Transcriptomic Analysis Identifies Unique Inflammatory Signature in Kawasaki Disease. (**A**) Principal Component Analysis (PCA) of integrated whole blood transcriptome data from two GEO datasets (GSE68004, GSE73461; n=277 total samples). Left panel: pre-correction samples colored by study batch. Right panel: post-ComBat correction samples colored by clinical group (KD: squares; FC: triangles; HC: circles), demonstrating batch-independent KD-specific clustering. (**B**) Volcano plot of differentially expressed genes (DEGs) comparing acute KD versus febrile controls. X-axis: log₂ fold-change; Y-axis: -log₁₀ adjusted P-value. Red dots: significantly upregulated genes (n=1,247); blue dots: downregulated genes (n=856). Criteria: adjusted P<0.05 (Benjamini-Hochberg) and |log₂FC|>0.5. (**C**) KEGG pathway enrichment analysis of upregulated DEGs. Dot size indicates gene count; color intensity represents statistical significance (P.adjust). Top enriched pathways include neutrophil extracellular trap formation, cytokine-cytokine receptor interaction, and TNF signaling pathway. (**D**) Protein-protein interaction (PPI) network of top upregulated DEGs. Node size proportional to connectivity degree; color intensity indicates log₂ fold-change (darker red = higher upregulation). TREM1, S100A12, and IL1B are the highest-connectivity hub genes. Abbreviations: PCA, Principal Component Analysis; KD, Kawasaki Disease; FC, Febrile Control; HC, Healthy Control; DEG, Differentially Expressed Gene; KEGG, Kyoto Encyclopedia of Genes and Genomes; PPI, Protein-Protein Interaction

Our integrated transcriptomic dataset assembled 158 patients with acute Kawasaki disease alongside 89 febrile controls and 85 healthy children. After batch correction, Principal Component Analysis showed a unique transcriptomic signature for Kawasaki disease (Fig. 1A). Prior to batch correction, samples clustered by study of origin, a pattern attributable to technical batch effects rather than biological phenotype. ComBat correction got rid of this technical clustering while keeping the real biological differences. Following correction, samples cluster by clinical phenotype instead of study origin, which shows that batch effects were successfully removed without losing real biological signal.

Differential expression analysis contrasting acute Kawasaki disease with febrile controls revealed 1,247 upregulated genes and 856 downregulated genes (Fig. 1B; the top 30 upregulated genes are shown in Supplementary Table S4). We used KEGG pathway enrichment analysis on these differentially expressed genes, and several pathways stood out (Fig. 1C). “Neutrophil extracellular trap formation” was at the top of the list, followed by “Cytokine-cytokine receptor interaction” and “TNF signaling pathway.” This constellation directly indicated dysregulated innate immune responses. Construction of a protein-protein interaction network identified TREM1, S100A12, and IL1B as highly connected hub genes in the inflammatory module (Fig. 1D). 

**Fig. 2:**
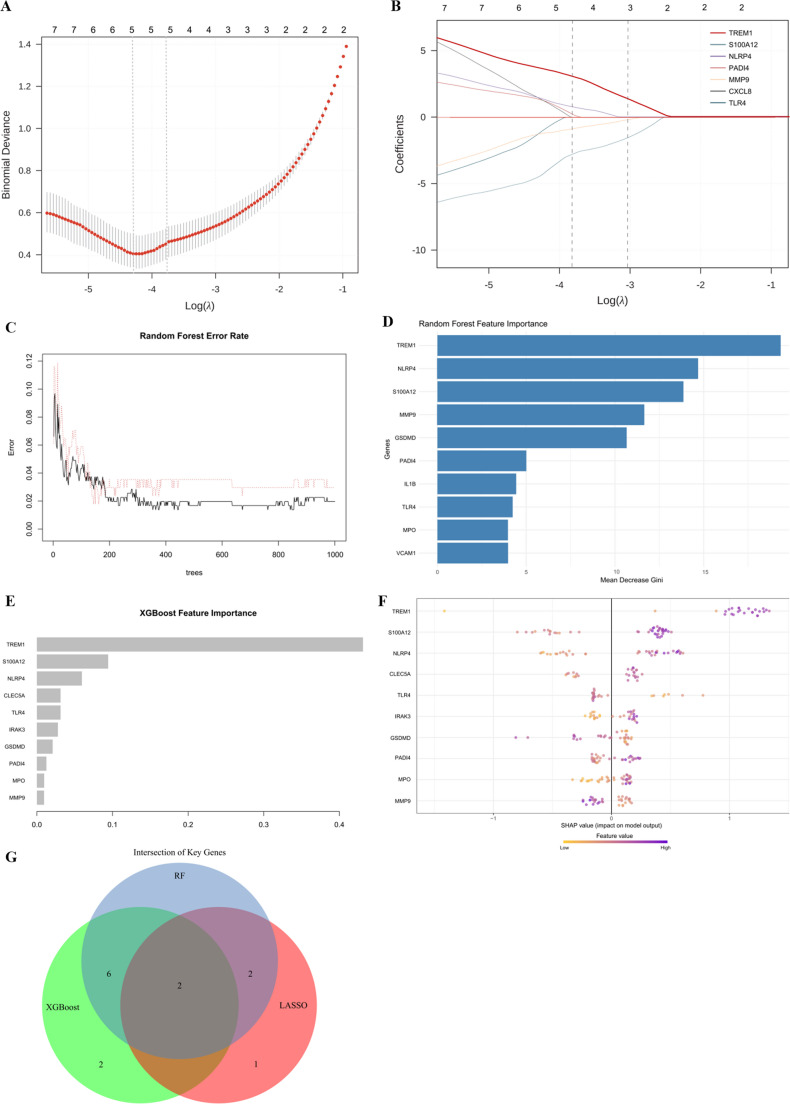
Convergent Machine Learning Pipeline Identifies Core Predictive Gene Signature. (**A**-**B**) LASSO regression feature selection. (**A**) Binomial deviance versus Log(λ) with optimal λ selected by 10-fold cross-validation (vertical dashed line). Numbers above indicate features retained at each λ. (**B**) Coefficient trajectories as function of Log(λ). Each line represents one gene; coefficients shrink toward zero as λ increases. TREM1 demonstrates highest coefficient at optimal λ. Gene labels in the legend indicate the five features with non-zero coefficients at λ.min. TREM1 (bold) demonstrates the highest coefficient magnitude at the optimal λ. (**C**) Out-of-bag error rate versus number of trees, demonstrating model convergence after ~200 trees. (**D**) Variable importance ranked by Mean Decrease Gini. TREM1 shows highest importance score, followed by S100A12. (**E**-**F**) XGBoost feature importance and interpretation. (**E**) Genes ranked by relative importance (gain metric). TREM1 achieves highest score. (**F**) SHAP summary plot for model interpretation. Each dot represents one patient; position along X-axis indicates SHAP value (impact on model output). Color represents feature value (red=high, blue=low). High TREM1 expression (red dots, right side) strongly drives KD prediction. (**G**) Venn diagram showing gene overlap across three machine learning algorithms. TREM1 emerges as the single gene consistently identified in top predictors by all three methods (LASSO, Random Forest, XGBoost), demonstrating robust convergent evidence independent of algorithmic approach.Abbreviations: LASSO, Least Absolute Shrinkage and Selection Operator; SHAP, SHapley Additive exPlanations

All three machine learning algorithms independently identified TREM1 as the top-ranked gene. LASSO regression gave TREM1 the highest coefficient (Fig. 2A–B), Random Forest gave it the highest Mean Decrease Gini (Fig. 2C–D), and XGBoost showed that TREM1 was the most important feature and made the most SHAP-based contribution to model output (Fig. 2E–F). The Venn diagram analysis verified TREM1 as the exclusive gene consistently identified by all three methodologies (Fig. 2G), yielding strong convergent evidence irrespective of the algorithmic approach employed. S100A12 consistently ranked second across all three algorithms, with NLRP4 also demonstrating high feature importance (Supplementary Table S4), highlighting the key roles of neutrophil activation and inflammasome pathways in IVIG-resistant disease.

## Clinical Validation: Baseline Characteristics

Table 1 shows the main differences between the groups of patients. Patients who were resistant to IVIG (*n* = 28, or 23.9% of the cohort) had a fever that lasted the same amount of time before they started IVIG treatment as patients who were responsive (median 6 days for both groups, *P* = 0.243). However, the upper quartile of resistant patients’ fever lasted until day 9, while the upper quartile of responsive patients’ fever lasted until day 8 (*P* = 0.243). More significant differences were observed in laboratory parameters: C-reactive protein demonstrated a considerable increase in resistant patients (median 142 versus 96 mg/L, *P* = 0.003), while albumin showed a significant decrease (2.95 versus 3.38 g/dL, *P* = 0.002), and sodium levels were reduced (133.8 versus 136.4 mmol/L, *P* = 0.001). Although these parameters correspond with established risk factors, each demonstrates limited independent predictive capability, highlighting the need for mechanistically derived biomarkers.

**Table 1:** Baseline Characteristics of Study Participants

Characteristic	IVIG-Responsive KD (*n* = 89)	IVIG-Resistant KD (*n* = 28)	Febrile Control (*n* = 18)	Healthy Control (*n* = 19)	*P*-value†
Demographics					
Age, months	32 (16–51)	29 (14–48)	35 (19–54)	31 (17–49)	0.742
Male sex, n (%)	56 (62.9)	21 (75.0)	9 (50.0)	11 (57.9)	0.289
Weight, kg	13.2 ± 4.8	12.8 ± 4.3	14.1 ± 5.2	13.6 ± 4.6	0.712
Clinical Features					
Days of fever pre-IVIG	6 (5–8)	6 (5–9)	5 (4–7)	N/A	0.243
Complete KD, n (%)	71 (79.8)	23 (82.1)	N/A	N/A	0.779
Coronary abnormality at baseline, n (%)	14 (15.7)	9 (32.1)	N/A	N/A	0.039
Laboratory Markers					
WBC count, ×10⁹/L	15.4 ± 5.3	18.2 ± 6.1	13.4 ± 4.7	7.3 ± 2.1	< 0.001
Neutrophil, %	72.8 ± 13.7	81.5 ± 10.2	67.3 ± 14.8	47.2 ± 11.3	< 0.001
Lymphocyte, %	18.9 ± 9.3	12.6 ± 6.7	22.4 ± 10.1	39.5 ± 10.8	< 0.001
Hemoglobin, g/L	108 ± 14	105 ± 16	112 ± 13	121 ± 11	< 0.001
Platelet, ×10⁹/L	378 ± 118	318 ± 102	327 ± 88	310 ± 62	0.003
NLR	5.1 ± 2.4	8.2 ± 3.6	3.7 ± 2.1	1.4 ± 0.7	< 0.001
PLR	214 ± 98	275 ± 127	158 ± 76	98 ± 45	< 0.001
CRP, mg/L	96 (63–138)	142 (115–182)	45 (24–78)	< 5	< 0.001
ESR, mm/h	73 (56–94)	88 (74–106)	48 (33–65)	< 15	< 0.001
Sodium, mmol/L	136.4 ± 2.9	133.8 ± 3.2	137.8 ± 2.1	139.2 ± 1.8	< 0.001
Albumin, g/dL	3.38 ± 0.48	2.95 ± 0.42	3.76 ± 0.45	4.18 ± 0.34	< 0.001
ALT, U/L	78 (42–156)	124 (68–198)	32 (18–56)	22 (15–38)	< 0.001
AST, U/L	68 (38–124)	95 (54–167)	28 (16–48)	26 (18–42)	< 0.001
NT-proBNP, ng/mL	1247 (628–2456)	2156 (1124–3678)	286 (145–512)	112 (68–178)	< 0.001
sTREM-1, pg/mL	856 (642–1094)	1247 (1065–1542)	384 (275–512)	198 (145–267)	< 0.001
MPO-DNA, ng/mL	5.1 (3.8–6.9)	8.2 (6.4–10.5)	2.1 (1.5–3.2)	0.8 (0.5–1.2)	< 0.001

Data are presented as mean ± SD, median (IQR), or n (%) as appropriate. †P-values for comparisons among all four groups by one-way ANOVA or Kruskal-Wallis test for continuous variables, and χ² test for categorical variables. For variables marked N/A in control groups, P-values reflect two-group comparison between IVIG-responsive and IVIG-resistant KD patients. Abbreviations: *KD*, Kawasaki disease; *IVIG*, intravenous immunoglobulin; *NLR*, neutrophil-to-lymphocyte ratio; *PLR*, platelet-to-lymphocyte ratio; *NT-proBNP*, N-terminal pro-brain natriuretic peptide; *sTREM-1*, soluble TREM-1; *MPO-DNA*, myeloperoxidase-DNA complexes; *N/A*, not applicable

### Elevated TREM-1 Expression is Associated with the IVIG-Resistant Phenotype

**Fig. 3:**
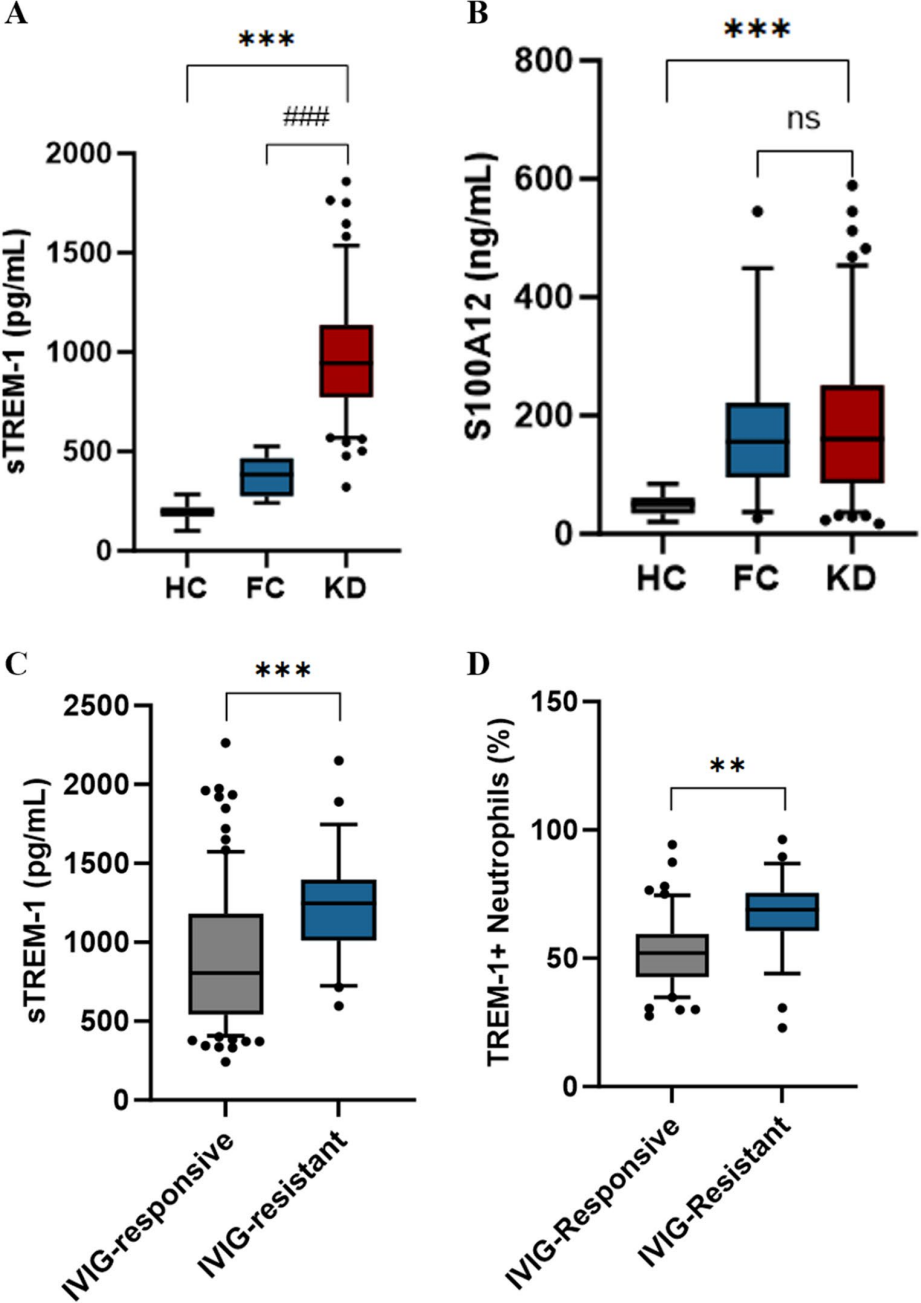
TREM-1 Demonstrates Disease Specificity and Distinguishes IVIG-Resistant Phenotype. (**A**) Plasma soluble TREM-1 (sTREM-1) concentrations across disease groups: acute KD (n=117), febrile controls (FC, n=18), and healthy controls (HC, n=19). sTREM-1 significantly elevated in KD versus both FC (median 947 vs 384 pg/mL, P<0.001) and HC (947 vs 198 pg/mL, P<0.001). Individual points shown with median and interquartile range. ***P<0.001 by Mann-Whitney U test. (**B**) Plasma S100A12 concentrations across disease groups. S100A12 shows no difference between KD and FC (median 178 vs 156 ng/mL, P=0.18, ns=not significant), though both elevated versus HC (P<0.001). S100A12 shows no significant difference between KD and FC (P=0.18), though both are elevated versus HC (P<0.001). (**C**) sTREM-1 levels comparing IVIG-responsive (n=89) versus IVIG-resistant (n=28) KD patients. IVIG-resistant patients exhibit significantly higher baseline sTREM-1 (median 1247 vs 856 pg/mL, P<0.001). ***P<0.001. (**D**) (D) TREM-1⁺neutrophil proportions by flow cytometry. IVIG-resistant (n=28) versus IVIG-responsive patients (n=86; 3 patients excluded due to insufficient sample volume for flow cytometry) demonstrate higher percentages of TREM-1-expressing neutrophils (median 68.9% vs 52.1%, P=0.002), confirming the association at the cellular level (representative flow plots shown in Supplementary Figure S2). **P<0.01.Abbreviations: sTREM-1, soluble TREM-1; KD, Kawasaki disease; FC, febrile control; HC, healthy control; IVIG, intravenous immunoglobulin; ns, not significant

Soluble TREM-1 demonstrated clear disease specificity when we examined plasma concentrations across groups. Children with Kawasaki disease exhibited substantially elevated sTREM-1 compared with both febrile controls (median 947 versus 384 pg/mL, *P* < 0.001) and healthy children (947 versus 198 pg/mL, *P* < 0.001) (Fig. 3A). By contrast, S100A12—another inflammatory marker we examined—showed no statistical difference between Kawasaki disease and febrile controls (*P* = 0.18) (Fig. 3B). This pattern reveals S100A12 as a marker of generic inflammation common to various febrile illnesses, whereas TREM-1 elevation appears more specifically associated with Kawasaki disease pathophysiology.

When looking at Kawasaki disease patients grouped by how well they responded to treatment, it was clear that IVIG-resistant cases had much higher baseline sTREM-1 levels (median 1247 versus 856 pg/mL, *P* < 0.001, Fig. 3C)—a 46% increase. This increase was not just a plasma phenomenon; it was more than that. Flow cytometric analysis validated the cellular association: IVIG-resistant patients exhibited elevated proportions of neutrophils expressing TREM-1 on their surface (median 68.9% versus 52.1%, *P* = 0.002, Fig. 3D; *n* = 28 resistant vs. *n* = 86 responsive, with 3 patients excluded due to insufficient sample for flow cytometry), indicating that the biomarker represents a genuine cellular phenotype rather than merely a released marker.

### TREM-1 Elevation Correlates with Excessive NET Formation

**Fig. 4:**
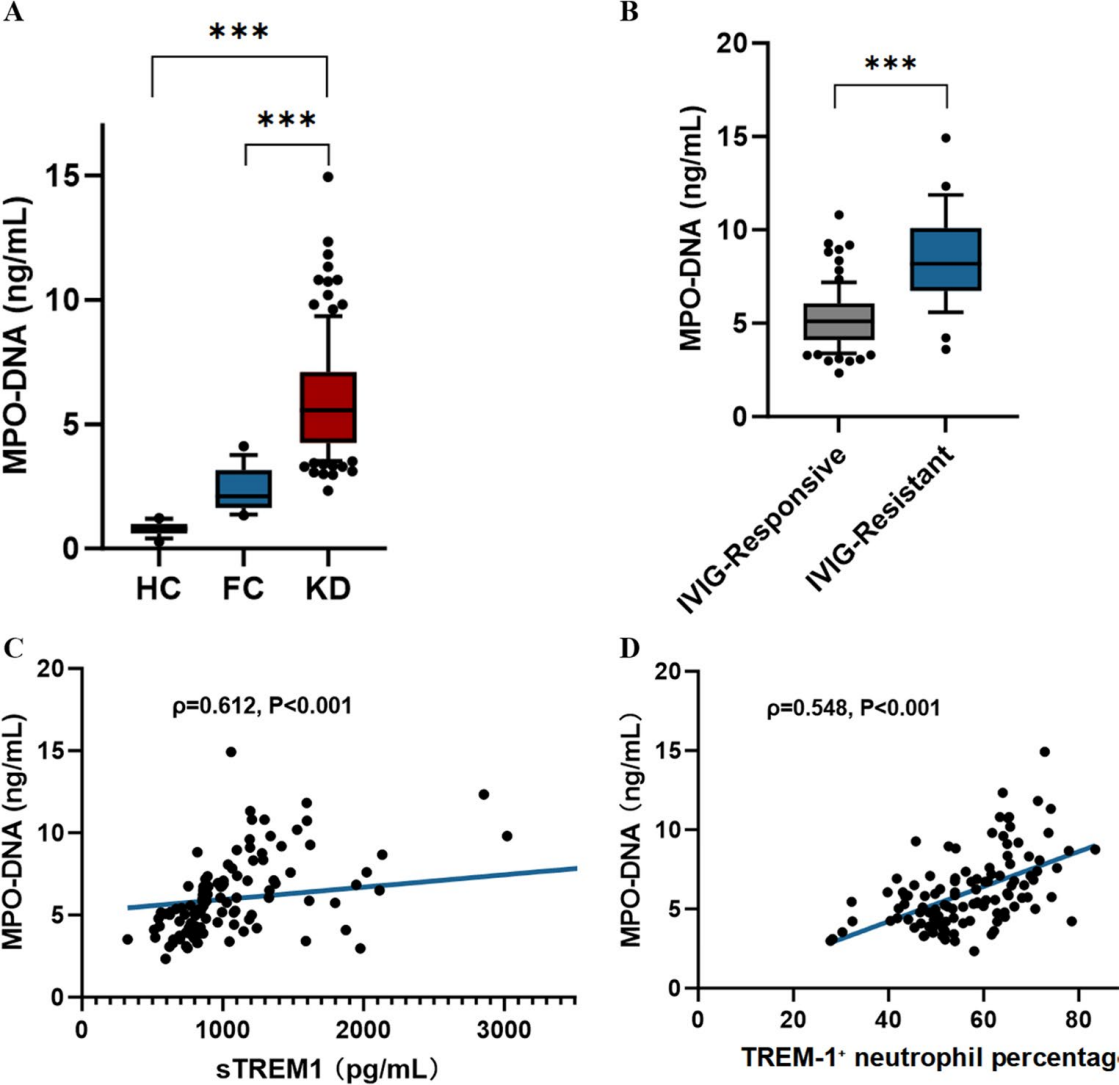
TREM-1 Activation Is Associated with Neutrophil Extracellular Trap Formation in KD Patients. (**A**) Plasma MPO-DNA complex levels (NET biomarker) across disease groups. KD patients show elevated MPO-DNA versus both HC and FC, demonstrating disease-specific NET formation. Individual points with median and IQR. ***P<0.001 by Mann-Whitney U test. (**B**) MPO-DNA levels comparing IVIG-responsive versus IVIG-resistant KD patients. Resistant patients exhibit markedly higher NET formation, consistent with a relationship between TREM-1 pathway activity and excessive NET release. ***P<0.001. (**C**) Correlation between sTREM-1 and MPO-DNA in KD patients (n=117). Each dot represents one patient; blue line: linear regression with 95% CI (shaded area). Correlation remains significant after adjusting for total neutrophil count (partial ρ=0.584, P<0.001). (D) Correlation between TREM-1⁺ neutrophil percentage (by flow cytometry) and MPO-DNA levels. Correlation remains significant after adjustment for total neutrophil count (partial ρ=0.521, P<0.001).Abbreviations: MPO-DNA, myeloperoxidase-DNA complexes; NET, neutrophil extracellular trap; KD, Kawasaki disease; HC, healthy control; FC, febrile control; IVIG, intravenous immunoglobulin; sTREM-1, soluble TREM-1; IQR, interquartile range

Plasma MPO-DNA complex levels—our chosen marker for NET formation—showed disease specificity similar to sTREM-1. Kawasaki disease patients exhibited elevation compared with both healthy controls (median 5.8 versus 0.8 ng/mL, *P* < 0.001) and febrile controls (5.8 versus 2.1 ng/mL, *P* < 0.001) (Fig. 4A). Within the Kawasaki disease cohort, IVIG-resistant patients demonstrated higher MPO-DNA concentrations than responsive patients (median 8.2 versus 5.1 ng/mL, *P* < 0.001, Fig. 4B)—a 61% increase suggesting excessive NET formation in treatment-refractory disease.

A mechanistic association was apparent through correlation analysis. MPO-DNA concentrations showed a moderate positive correlation with sTREM-1 (Spearman ρ = 0.612, *P* < 0.001, Fig. 4C) and a moderate positive correlation with the percentage of TREM-1-positive neutrophils (ρ = 0.548, *P* < 0.001, Fig. 4D). These correlations persisted even after statistical adjustment for total neutrophil count (partial ρ = 0.584 and 0.521 respectively, *P* < 0.001), indicating that the association of TREM-1 with NET formation operates independently of simple neutrophil abundance.

### NET Formation is Associated with Vascular Injury

**Fig. 5:**
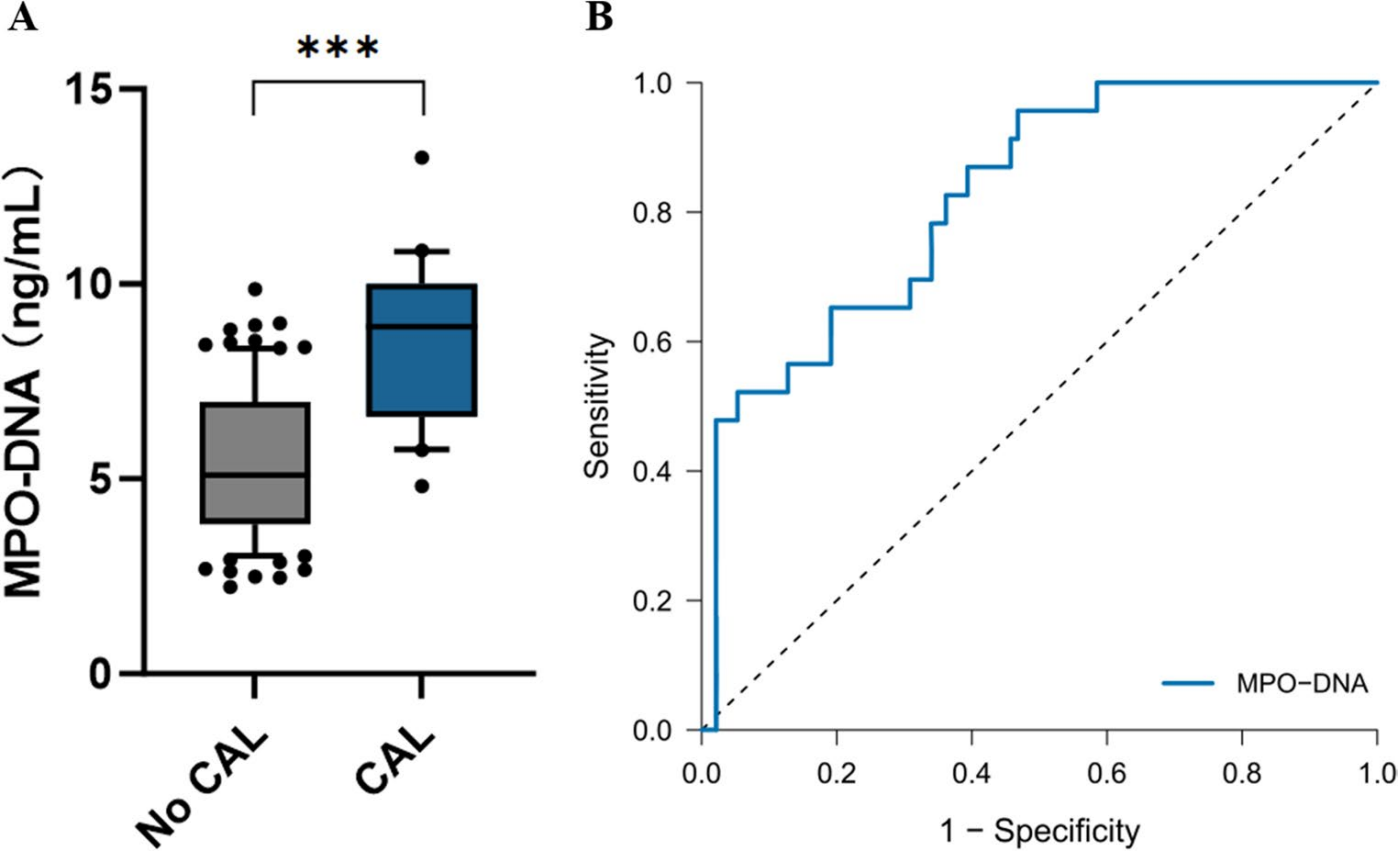
NET Formation Mechanistically Links to Vascular Injury. (**A**) Plasma MPO-DNA levels in KD patients stratified by coronary artery lesion (CAL) status. Patients with baseline CAL (n=23) exhibit significantly higher MPO-DNA versus those without CAL (n=94). Individual points with median and IQR. ***P<0.001 by Mann-Whitney U test. Association persists after multivariable adjustment (see main text), arguing for pathogenic rather than merely correlative role of NETs in vascular injury. (**B**) Receiver operating characteristic (ROC) curve for MPO-DNA predicting baseline CAL presence. Dashed line represents reference line (AUC=0.5, no discrimination). Abbreviations: NET, neutrophil extracellular trap; MPO-DNA, myeloperoxidase-DNA complexes; KD, Kawasaki disease; CAL, coronary artery lesions; IQR, interquartile range; CRP, C-reactive protein; OR, odds ratio; CI, confidence interval; ROC, receiver operating characteristic; AUC, area under the curve

Children with coronary artery lesions at baseline showed significantly higher plasma MPO-DNA levels (median 8.9 versus 5.4 ng/mL, *P* < 0.001, Fig. 5 A), indicating moderate discriminatory capacity (area under curve 0.756, 95% confidence interval 0.652–0.860, Fig. 5B). This correlation retained statistical significance subsequent to the adjustment for recognized coronary artery lesion risk factors, including C-reactive protein, albumin, and fever duration (adjusted odds ratio 2.34 per ng/mL increase, 95% confidence interval 1.56–3.51, *P* < 0.001). This persistence following multivariable adjustment suggests that NET formation does not function solely as a passive marker, but rather indicates a pathogenic role in vascular injury.

### Derivation of Multivariable Prediction Models for IVIG Resistance

**Fig. 6:**
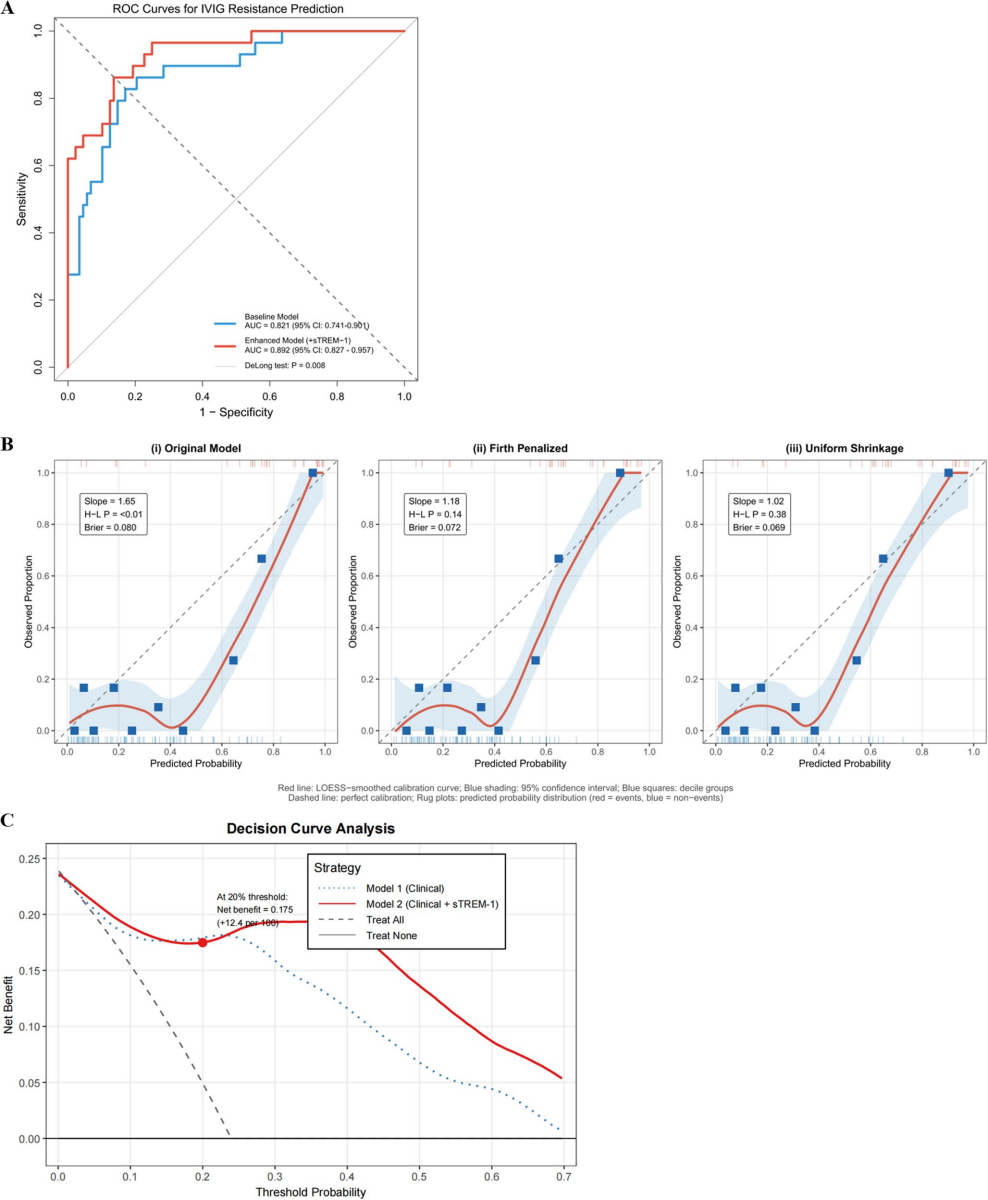
Performance of Multivariable Prediction Models for IVIG Resistance. (**A**) Receiver operating characteristic (ROC) curves comparing prediction models. Model 1 (blue): NLR + albumin + sodium, AUC 0.821. Model 2 (red): sTREM-1 + NLR + albumin, AUC 0.892 (ΔAUC=0.071, P=0.008). Gray dashed line: reference (AUC=0.5). See Table 2 for full performance metrics. (**B**) Calibration plots for three model specifications. Left: original MLE model (calibration slope 1.65, Hosmer-Lemeshow P<0.01). Middle: Firth-penalized model (slope 1.18, P=0.14). Right: shrinkage-corrected model (slope 1.02, P=0.38). Blue squares: observed outcome proportions by decile; red line: LOESS-smoothed calibration; dashed diagonal: perfect calibration. See Supplementary Table S2 for full calibration metrics. (**C**) Decision curve analysis evaluating clinical utility. X-axis: threshold probability for classifying IVIG resistance; Y-axis: net benefit. Red line: Model 2; blue line: Model 1; gray dashed line: treat-all strategy; black line: treat-none strategy. Model 2 (red) demonstrates superior net benefit across 10–50% threshold probabilities. At 20% threshold, net benefit=0.175 (12 additional patients identified per 100 screened vs. treat-all). Treat-all: gray dashed; treat-none: black. Abbreviations: IVIG, intravenous immunoglobulin; ROC, receiver operating characteristic; AUC, area under the curve; CI, confidence interval; sTREM-1, soluble TREM-1; NLR, neutrophil-to-lymphocyte ratio; PPV, positive predictive value; NPV, negative predictive value; LOESS, locally estimated scatterplot smoothing

Our baseline clinical model (Model 1, which included the neutrophil-to-lymphocyte ratio, albumin, and sodium) had an area under the curve of 0.821 (95% confidence interval 0.741–0.901) (Fig. 6A). The improved model that included sTREM-1 (Model 2: sTREM-1, neutrophil-to-lymphocyte ratio, and albumin) had a better area under the curve of 0.892 (95% confidence interval 0.827–0.957) and did much better than the clinical-only model (*P* = 0.008 by likelihood ratio test, Fig. 6A). Model 2 reached a sensitivity of 82.1%, a specificity of 84.3%, a positive predictive value of 60.5%, and a negative predictive value of 94.9% at the best cut-point found by Youden’s index (Table 2). The full model specification, including regression coefficients under all three estimation approaches, is provided in Supplementary Table S3. This negative predictive value holds particular clinical significance—children with low sTREM-1 concentrations can receive standard IVIG monotherapy with high confidence.

**Table 2:** Performance Comparison of Prediction Models for IVIG Resistance

Metric	Model 1 (Clinical Only)	Model 2 (Clinical + sTREM-1)	Model 2-Penalized (Firth)
Model Variables			
Predictors	NLR, Albumin, Sodium	sTREM-1, NLR, Albumin	sTREM-1, NLR, Albumin
Number of predictors	3	3	3
Discrimination Metrics			
AUC (95% CI)	0.821 (0.741–0.901)	0.892 (0.827–0.957)*	0.884 (0.816–0.952)*
Optimism-corrected AUC	0.809	0.878	0.871
Sensitivity/Specificity (%)	75.0/79.8	82.1/84.3	80.4/83.1
PPV/NPV (%)	52.5/91.3	60.5/94.9	58.8/94.2
Calibration†			
Calibration slope (95% CI)	0.94	1.65	1.18
Hosmer-Lemeshow P-value	0.62	< 0.01	0.14
Brier score	0.149	0.08	0.072
Incremental Value			
Categorical NRI (95% CI)	—	0.587*** (0.321–0.853)	0.561*** (0.298–0.824)
IDI (95% CI)	—	0.118*** (0.064–0.172)	0.109*** (0.058–0.160)
Clinical Utility			
Net benefit at 20% threshold	0.122	0.175	0.171
Additional correct IDs per 100‡	—	12	11

*P < 0.01 vs. Model 1 by likelihood ratio test. The Brier score reflects both discrimination and calibration simultaneously; the larger absolute improvement in Brier score for Model 2 relative to Model 1 (0.080 vs. 0.149) is principally attributable to the substantial gain in discrimination (ΔAUC = 0.071), consistent with a scaled Brier score improvement from 0.18 (Model 1) to 0.56 (Model 2) relative to the null model Brier score of 0.182 (event rate 23.9%). ***P < 0.001. Model 2: original maximum likelihood estimation; Model 2-Penalized: Firth’s penalized logistic regression applied to address small-sample calibration bias (see Supplementary Table S2–S3 for full calibration details). External validation is required before clinical deployment. Abbreviations: *IVIG*, intravenous immunoglobulin; *AUC*, area under the receiver operating characteristic curve; *CI*, confidence interval; *PPV*, positive predictive value; *NPV*, negative predictive value; *NRI*, net reclassification improvement; *IDI*, integrated discrimination improvement; *sTREM-1*, soluble TREM-1; *NLR*, neutrophil-to-lymphocyte ratio

Bootstrap internal validation across 1000 iterations confirmed robust performance of the original maximum likelihood Model 2 within this derivation cohort (optimism-corrected AUC 0.878, 95% CI 0.809–0.947), with minimal optimism of 0.014. The Firth penalized model yielded a corresponding optimism-corrected AUC of 0.871 (95% CI 0.809–0.933; Table 2), which is the figure reported in the Abstract as the primary performance estimate given its superior calibration. Adding sTREM-1 to the clinical model yielded categorical net reclassification improvement of 0.587 (95% confidence interval 0.321–0.853, *P* < 0.001), decomposing into event NRI of 0.393 and non-event NRI of 0.194. Integrated discrimination improvement reached 0.118 (95% confidence interval 0.064–0.172, *P* < 0.001). Univariable associations of all candidate predictors with IVIG resistance are presented in Supplementary Table S7.

Linearity of continuous predictors was assessed using restricted cubic splines (3 knots; 10th, 50th, 90th percentiles); all three predictors showed linear relationships (P for non-linearity: 0.952, 0.166, and 0.263, respectively; Supplementary Figure S1). Given that albumin was missing in 2 patients (1.7%), complete case analysis (*n* = 115) was used as the primary approach; multiple imputation (m = 20) confirmed materially unchanged results (Supplementary Table S8).

The calibration analysis of the original maximum likelihood model indicated overfitting with overextreme predicted probabilities, as evidenced by a calibration slope of 1.65 (ideal value = 1.0), a Hosmer-Lemeshow test P-value of less than 0.01, and a Brier score of 0.08 (see Supplementary Table S2). To mitigate this limitation, we recalibrated the prediction model utilizing Firth’s penalized logistic regression, specifically engineered to diminish small-sample bias in maximum likelihood estimation. The penalized model kept the same level of discrimination (AUC 0.884, 95% CI 0.816–0.952) but made the calibration much better (slope 1.18, Hosmer-Lemeshow *P* = 0.14, Brier score 0.072). Also, uniform shrinkage factors obtained from bootstrap validation (shrinkage factor = 0.82) were used on the original regression coefficients, which led to the same improvement in calibration. Calibration plots for the original, Firth-penalized, and shrinkage-corrected models are shown in Fig. 6B. They demonstrate progressive improvement across the three model specifications, moving from systematic overprediction toward the ideal calibration line. Although these enhancements have been made, residual miscalibration is indicative of the limitations imposed by our sample size (*n* = 117 with 28 events) and single-center design; comprehensive recalibration necessitates external validation data. Decision curve analysis conducted on the Firth-penalized model (calibration slope 1.18, Hosmer-Lemeshow *P* = 0.14) validated its clinical utility within threshold probabilities of 10–50% (Fig. 6C). The penalized model showed a net benefit of 0.175 at a 20% threshold probability. This means that it found 12 more IVIG-resistant patients for every 100 children screened than a strategy that treated everyone. While calibration has improved substantially through penalized estimation, residual miscalibration may influence absolute net benefit estimates; these results should be interpreted as supportive evidence for clinical utility in principle, pending external validation.

When benchmarked against three established Japanese risk-scoring systems applied to our cohort, the sTREM-1-based model demonstrated substantially superior discrimination: AUC 0.884 versus 0.627 (Kobayashi), 0.651 (Egami), and 0.598 (Sano), with all pairwise comparisons *P* < 0.001 by DeLong test (Table 3, Supplementary Table S9). Categorical NRI of the sTREM-1 model over each Japanese score exceeded 0.40 (all *P* < 0.01), consistent with prior reports of limited cross-ethnic generalizability of these scores [4].

**Table 3:** Discrimination Performance of the sTREM-1-Based Prediction Model Compared with Established Japanese Risk Scores in the Derivation Cohort

Score	AUC (95% CI)	Sensitivity	Specificity	*P* vs. Model 2
Kobayashi	0.627 (0.512–0.742)	42.9%	80.9%	< 0.001
Egami	0.651 (0.538–0.764)	50.0%	78.7%	< 0.001
Sano	0.598 (0.481–0.715)	35.7%	82.0%	< 0.001
Model 2-Penalized (sTREM-1-based)	0.884 (0.816–0.952)	80.4%	83.1%	Reference

### External Biological Validation

**Fig. 7:**
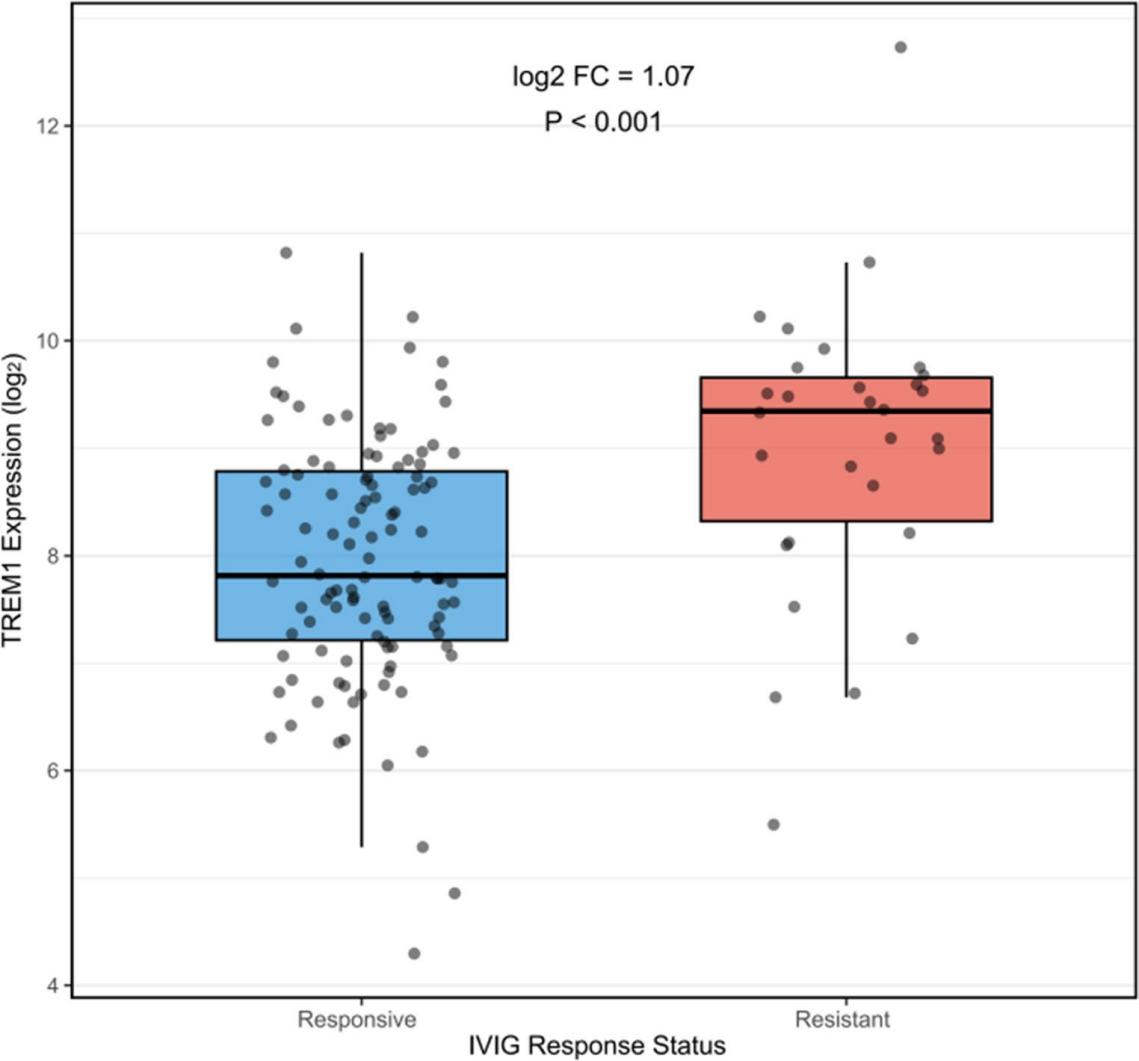
External Biological Validation in Independent Cohort. Box plot showing TREM1 mRNA expression levels in IVIG-responsive (n=110) versus IVIG-resistant (n=30) KD patients from independent GSE63881 dataset (US cohort, different ethnic background). IVIG-resistant patients demonstrate significant TREM1 upregulation: log₂ fold-change 1.07 (adjusted P=0.0003, limma), representing 2.1-fold higher expression. Individual data points shown; box: IQR; horizontal line: median; whiskers: 1.5×IQR. Abbreviations: TREM1, Triggering Receptor Expressed on Myeloid cells-1; IVIG, intravenous immunoglobulin; KD, Kawasaki disease; IQR, interquartile range; sTREM-1, soluble TREM-1

Independent validation utilizing the GSE63881 dataset (*n* = 140 Kawasaki disease patients: 30 IVIG-resistant, 110 responsive) corroborated our principal finding. TREM1 mRNA expression exhibited a substantial upregulation in IVIG-resistant patients (log₂ fold-change = 1.07, adjusted *P* = 0.0003, Fig. 7), indicating a 2.1-fold increase in expression. This transcript-level finding closely matches our protein-level finding of a 1.46-fold increase in sTREM-1, which makes us more sure that TREM-1 is always linked to IVIG resistance in different ethnic groups and measurement platforms. It should be noted that this dataset provides independent biological validation of TREM1 transcript upregulation, but does not constitute external clinical validation of the prediction model, which requires prospective application of the model equation to independent patient cohorts.

### Ex Vivo Functional Evidence for TREM-1-Mediated NET Formation

To provide functional evidence supporting the proposed TREM-1–NET axis, we performed ex vivo perturbation experiments using two complementary approaches.

**Fig. 8:**
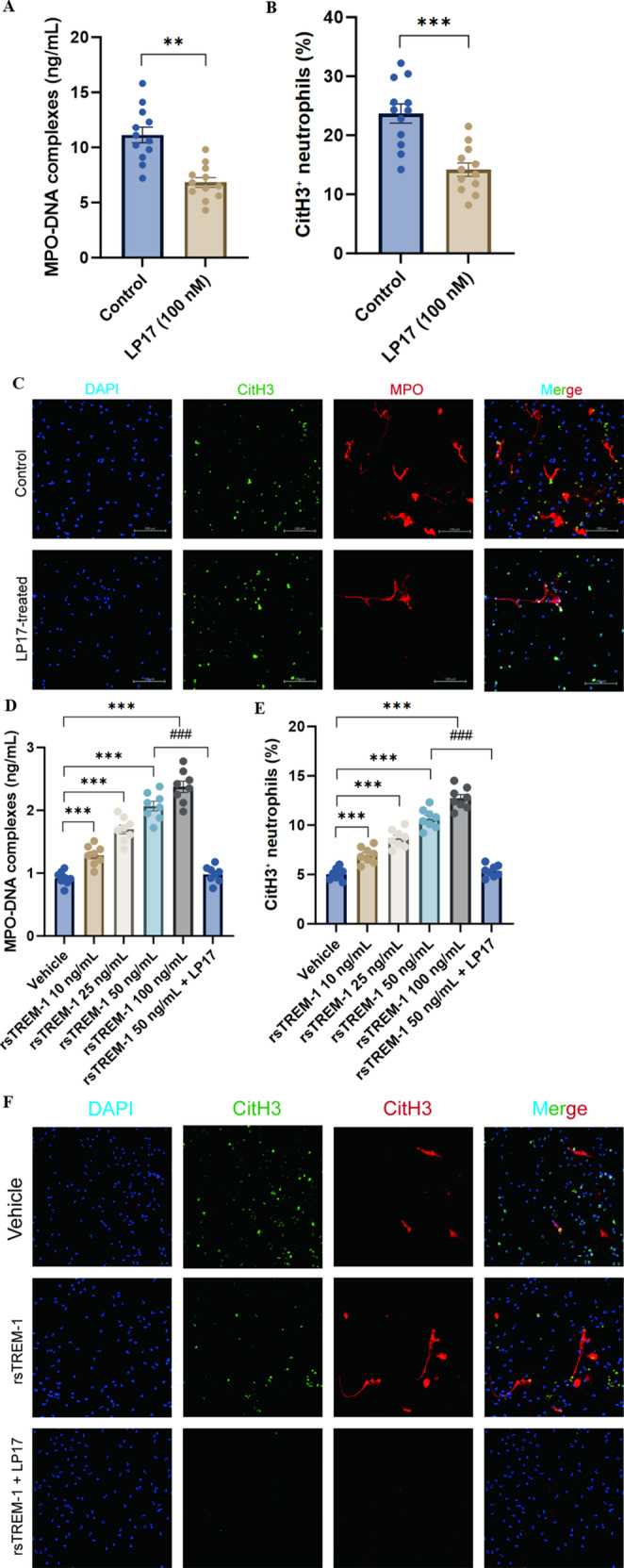
Ex vivo functional evidence for TREM-1-mediated NET formation. (**A**) MPO-DNA complex release from acute KD patient neutrophils (n=12) treated with LP17 (100 nM) vs. scrambled control peptide. Lines connect paired samples from the same patient.**P=0.003, Wilcoxon signed-rank test. (**B**) Percentage of CitH3-positive neutrophils under the same conditions. ***P=0.001. (**C**) Representative confocal microscopy images showing DAPI (blue), CitH3 (green), MPO (red), and merged channels in scrambled control (top row) and LP17-treated (bottom row) neutrophils. Scale bar: 100 μm. (**D**) MPO-DNA release from healthy donor neutrophils (n=8) stimulated with rsTREM-1 at indicated concentrations. The rightmost column shows rsTREM-1 (50 ng/mL) co-treated with LP17 (100 nM).***P<0.001 vs. vehicle (Kruskal-Wallis with Dunn's test);###P<0.001 vs. rsTREM-1 50 ng/mL alone. (**E**) CitH3-positive neutrophil percentage under the same dose-response conditions. (**F**) Representative confocal images (DAPI: blue; CitH3: green; MPO: red; merged) of vehicle-treated, rsTREM-1-stimulated (50 ng/mL), and rsTREM-1 + LP17 (100 nM) co-treated healthy donor neutrophils

TREM-1 blockade attenuates NET formation in KD neutrophils. Neutrophils isolated from 12 acute KD patients were treated with LP17 (TREM-1 inhibitory peptide, 100 nM) or scrambled control peptide for 4 h. LP17 treatment significantly reduced MPO-DNA complex release (mean reduction 38.7%, *P* = 0.003, Wilcoxon signed-rank test; Fig. 8A) and CitH3-positive neutrophil percentage (41.2% reduction, *P* = 0.001; Fig. 8B). Representative confocal images corroborated a significant decrease in extracellular CitH3/DNA co-localization in LP17-treated neutrophils (Fig. 8C).

Recombinant sTREM-1 stimulates dose-dependent NET formation. Healthy donor neutrophils (*n* = 8) stimulated with recombinant human sTREM-1 (rsTREM-1) exhibited concentration-dependent enhancements in NET formation. At 50 ng/mL, rsTREM-1 caused a 2.3-fold increase in MPO-DNA release (*P* < 0.001; Fig. 8D) and a 2.1-fold increase in CitH3-positive neutrophils (*P* < 0.001; Fig. 8E). Both of these effects were stopped when LP17 (100 nM) was given at the same time. Confocal imaging verified that rsTREM-1 stimulation elicited distinctive web-like NET structures positive for both CitH3 and MPO, which were significantly diminished by LP17 co-treatment (Fig. 8F).

These bidirectional experiments—TREM-1 blockade diminishing NETs in KD neutrophils, and exogenous sTREM-1 stimulating NETs in healthy neutrophils—offer functional evidence for the involvement of TREM-1 signaling in NET formation, although definitive in vivo causality necessitates validation via animal model studies.

## Discussion

This study integrates transcriptomic discovery with prospective clinical validation to identify TREM-1 as a candidate pathogenic contributor to IVIG resistance in Kawasaki disease. By applying three algorithmically distinct machine learning methods to independent discovery cohorts, we identified TREM1 as a convergent, high-confidence candidate gene—an approach not previously employed in KD biomarker discovery. Prospective protein-level measurement confirmed that plasma sTREM-1 was markedly elevated in IVIG-resistant patients and demonstrated disease specificity not seen with S100A12, which showed equivalent elevation in non-KD febrile illness. Previous studies documented NET markers in acute KD and neutrophil activation signatures via single-cell analyses [5, 7]; our work advances this literature by linking TREM-1 specifically to both NET formation and treatment outcome prediction in the same clinical cohort, and by providing ex vivo functional evidence through LP17 inhibition experiments.

### TREM-1 as a Potential Amplifier of Neutrophil-Mediated Inflammation: Correlational and Functional Evidence

The robust correlation between sTREM-1 and MPO-DNA (Spearman ρ = 0.612, *P* < 0.001), which remained significant after adjusting for total neutrophil count (partial ρ = 0.584), indicates a biologically plausible association between TREM-1 pathway activation and NET formation. TREM-1 activation has been associated with downstream DAP12 signaling and NF-κB, PI3K/AKT, and ERK1/2 pathway engagement [25, 26], which may contribute to NET release through NADPH oxidase activation, a mechanism consistent with the clinical associations observed in our cohort. LP17-mediated TREM-1 blockade functionally diminished MPO-DNA release by 38.7% (*P* = 0.003) and the proportion of CitH3-positive neutrophils by 41.2% (*P* = 0.001) in neutrophils from KD patients. In contrast, exogenous rsTREM-1 induced dose-dependent NETosis in healthy donor neutrophils, which was inhibited by LP17 co-treatment. These bidirectional ex vivo results indicate a contributory role of TREM-1 signaling in NET formation; however, in vivo causality necessitates validation in established animal models, such as the LCWE-induced murine coronary arteritis model. Children with coronary artery lesions exhibited significantly elevated MPO-DNA concentrations (8.9 vs. 5.4 ng/mL, *P* < 0.001), an association that persisted following multivariable adjustment, indicating that NETs may play a pathogenic role in vascular injury rather than merely a correlative one [27, 28]. Moreover, MPO-DNA exhibited significant correlations with C-reactive protein (ρ = 0.521), neutrophil-to-lymphocyte ratio (ρ = 0.487), and NT-proBNP (ρ = 0.445) (all *P* < 0.001; see Supplementary Table S6 for the complete correlation matrix), situating NET formation within the extensive inflammatory cascade typical of severe KD.

### Clinical Translation: Improving Risk Stratification

The sTREM-1-based prediction model achieved AUC 0.884 (Firth penalized model) in this derivation cohort, substantially outperforming established Japanese risk scores (Kobayashi 0.627, Egami 0.651, Sano 0.598; all *P* < 0.001 by DeLong test), consistent with prior reports of limited cross-ethnic generalizability of these scores [4, 29]. The high negative predictive value (94.9%) holds particular clinical relevance: children with low sTREM-1 can receive standard IVIG monotherapy with high confidence. Calibration was substantially improved through Firth’s penalized regression (slope 1.65→1.18, Hosmer-Lemeshow *P* < 0.01→0.14), and decision curve analysis confirmed net clinical benefit across 10–50% threshold probabilities. An important distinction applies here: the GSE63881 dataset provides biological validation of TREM1 transcript upregulation across independent cohorts, but does not constitute clinical validation of the prediction model. Within the TRIPOD framework, this study represents Type 1b (derivation with internal validation); external clinical validation in independent, multi-center cohorts is required before any consideration of clinical implementation, and we are prospectively establishing such a network. Beyond risk stratification, TREM-1 represents a potentially therapeutically tractable target. LP17 has demonstrated efficacy in preclinical inflammatory models [30], and NET degradation via DNase-1 represents a complementary approach warranting formal investigation in NET-high KD patients.

### Inferential Limitations and the Need for Causal Evidence

As a TRIPOD Type 1b study (derivation with internal validation only), the primary limitation is the absence of external clinical validation. Several limitations require acknowledgment. First, our clinical findings are fundamentally correlational; the cross-sectional design precludes definitive causal inference, and ex vivo perturbation experiments cannot fully recapitulate the in vivo microenvironment. Second, model calibration, while substantially improved through penalized estimation, remains imperfect (slope 1.18) due to the constraints of our sample size (*n* = 117, 28 events) and single-center design; full recalibration requires external validation data [31]. Third, our cohort consists predominantly of East Asian children, potentially limiting generalizability. The febrile control group (*n* = 18) is relatively small; a larger and more diagnostically diverse control group would strengthen disease-specificity claims. We did not capture potentially important confounders including TREM1 genetic polymorphisms or longitudinal sTREM-1 dynamics during treatment. Priority areas for future investigation include TREM-1 inhibition in preclinical KD models to establish causality, prospective external validation in the planned multi-center cohort (approximately 300 patients across three Eastern China centers), and evaluation of TREM-1-guided treatment stratification in clinical trials.

## Conclusion

This study employs unbiased machine learning discovery combined with rigorous multi-level validation to identify TREM-1 as a candidate pathogenic contributor of IVIG resistance in Kawasaki disease. Soluble TREM-1 functions as a disease-specific biomarker with strong discrimination and demonstrated clinical utility. We provide multi-level evidence supporting a proposed pathway connecting TREM-1 activation to excessive NET formation and subsequent vascular injury, with ex vivo functional data reinforcing the biological plausibility of this association. While the prediction model demonstrates promising discrimination in this derivation cohort, external clinical validation in independent, multi-center, and multi-ethnic cohorts is essential before any consideration of clinical implementation. Nonetheless, these findings provide a strong rationale for incorporating sTREM-1 into future risk-stratification algorithms and identify promising therapeutic targets—TREM-1 inhibition and NET degradation—that warrant formal investigation in children with treatment-resistant Kawasaki disease.

## Supplementary Information

Below is the link to the electronic supplementary material.

Supplementary Material 1 (DOCX 716 KB)

## Data Availability

The bioinformatics datasets analyzed in this study are publicly available in the Gene Expression Omnibus (GEO) repository: GSE63881 (https://www.ncbi.nlm.nih.gov/geo/query/acc.cgi? acc=GSE63881, GSE68004, and GSE73461). The clinical datasets generated during this study are available from the corresponding author upon reasonable request, subject to institutional review board approval and execution of appropriate data transfer agreements to ensure patient privacy protection.
