# Supplementary material for: TREM-1-Associated Neutrophil Extracellular Trap Formation is Linked to IVIG Resistance in Kawasaki Disease: A Convergent Transcriptomic and Prospective Validation Study
Source: J Clin Immunol. 2026 Apr 21;46(1):58. doi: 10.1007/s10875-026-02025-x (PMC13230363; doi:10.1007/s10875-026-02025-x)
Supplement: Supplementary file 1 — Supplementary Material 1 (DOCX 716 KB) [file 10875_2026_2025_MOESM1_ESM.docx]

# SUPPLEMENTARY MATERIALS

## SUPPLEMENTARY METHODS

### Detailed Statistical Analysis Procedures

#### 1. Sample Size Calculation and Power Analysis

Given the exploratory nature of biomarker discovery and the limited events-per-variable (EPV) ratio in our cohort (n=117, 28 IVIG-resistant events), we conducted post-hoc power analysis to assess the statistical power of our findings.

For logistic regression with three predictors:

- Observed AUC: 0.892
- Sample size: 117
- Events: 28 (23.9%)
- Estimated power: >80% for detecting OR ≥2.0

**EPV Considerations:**
Traditional guidelines suggest ≥10 events per variable (EPV≥10) for stable logistic regression models. Our model with 3 predictors yields EPV=28/3≈9.3, slightly below this threshold. To address this limitation, we implemented:

1. **Variable selection constraint:** Maximum 3-4 predictors to maintain EPV>7

2. **Bootstrap internal validation:** 1000 iterations to estimate optimism

3. **Penalized regression:** Applied shrinkage factors to regression coefficients

4. **Cross-validation:** Used in machine learning feature selection phase

These approaches align with recent guidelines (Riley et al., 2019; Vittinghoff & McCulloch, 2007) demonstrating that EPV<10 can be acceptable with appropriate statistical safeguards.

doi:10.1002/sim.7992

doi:10.1093/aje/kwk052

#### 2. Machine Learning Model Development

**LASSO Regression (Least Absolute Shrinkage and Selection Operator):**

# R code for LASSO implementation
library(glmnet)

# Prepare data matrix
x_matrix <- as.matrix(gene_expression_data)
y_response <- as.factor(ivig_resistance_status)

# 10-fold cross-validation to determine optimal lambda
cv_lasso <- cv.glmnet(x_matrix, y_response,
 family = "binomial",
 alpha = 1,
 nfolds = 10,
 type.measure = "class")

# Extract optimal lambda
lambda_min <- cv_lasso$lambda.min
lambda_1se <- cv_lasso$lambda.1se

# Fit final model with optimal lambda
lasso_model <- glmnet(x_matrix, y_response,
 family = "binomial",
 alpha = 1,
 lambda = lambda_min)

# Extract non-zero coefficients
lasso_coefficients <- coef(lasso_model, s = lambda_min)
selected_genes_lasso <- names(lasso_coefficients[lasso_coefficients != 0, ])

**Random Forest Classification:**

# R code for Random Forest implementation
library(randomForest)

# Set seed for reproducibility
set.seed(123)

# Train Random Forest model
rf_model <- randomForest(x = gene_expression_data,
 y = as.factor(ivig_resistance_status),
 ntree = 500,
 mtry = sqrt(ncol(gene_expression_data)),
 importance = TRUE,
 nodesize = 5)

# Extract variable importance
var_importance <- importance(rf_model, type = 2)
ranked_genes_rf <- rownames(var_importance)[order(var_importance[, "MeanDecreaseGini"],
 decreasing = TRUE)]

**XGBoost (Extreme Gradient Boosting):**

# R code for XGBoost implementation
library(xgboost)
library(SHAPforxgboost)

# Prepare data
dtrain <- xgb.DMatrix(data = as.matrix(gene_expression_data),
 label = ivig_resistance_status)

# Set hyperparameters (optimized via 5-fold CV)
params <- list(
 objective = "binary:logistic",
 eval_metric = "auc",
 max_depth = 6,
 eta = 0.1,
 gamma = 0,
 subsample = 0.8,
 colsample_bytree = 0.8,
 min_child_weight = 1
)

# 5-fold cross-validation for hyperparameter tuning
cv_results <- xgb.cv(params = params,
 data = dtrain,
 nrounds = 100,
 nfold = 5,
 early_stopping_rounds = 10,
 verbose = 0)

# Train final model
xgb_model <- xgb.train(params = params,
 data = dtrain,
 nrounds = cv_results$best_iteration)

# Calculate SHAP values
shap_values <- shap.values(xgb_model = xgb_model,
 X_train = as.matrix(gene_expression_data))

# Extract feature importance
importance_matrix <- xgb.importance(model = xgb_model)
ranked_genes_xgb <- importance_matrix$Feature

#### 3. Model Calibration Assessment

**Calibration Slope:**
Calibration slope quantifies the agreement between predicted probabilities and observed event rates. A slope of 1.0 indicates perfect calibration, while slope >1 indicates overprediction (predicted probabilities too extreme).

# Calculate calibration slope
library(rms)

# Fit logistic regression model
lrm_model <- lrm(IVIG_resistant ~ sTREM1 + NLR + Albumin,
 data = clinical_data)

# Extract calibration slope from validation
val_results <- validate(lrm_model, B = 1000)
calibration_slope <- val_results["Slope", "index.corrected"]

**Hosmer-Lemeshow Test:**
Groups patients by predicted probability deciles and compares observed vs. expected events in each group.

# Hosmer-Lemeshow test
library(ResourceSelection)

hoslem_test <- hoslem.test(clinical_data$IVIG_resistant,
 fitted(logistic_model),
 g = 10)

**Brier Score:**
Measures the mean squared difference between predicted probabilities and actual outcomes. Lower scores indicate better calibration (0 = perfect, 0.25 = uninformative).

# Calculate Brier score
brier_score <- mean((predicted_probabilities - actual_outcomes)^2)

**Linearity Assessment for Continuous Predictors**

The linearity assumption for each continuous predictor in the logistic regression model was assessed using restricted cubic splines (RCS) with 3 knots placed at the 10th, 50th, and 90th percentiles of the predictor distribution, as recommended by Harrell (2015). The likelihood ratio test comparing the nonlinear spline model against the linear model was used to assess departure from linearity. sTREM-1 and NLR showed approximately linear relationships with the log-odds of IVIG resistance (P=0.952、P=0.166 for non-linearity, respectively). Albumin demonstrated borderline non-linearity (P=0.263) but was retained in linear form given the limited sample size and marginal statistical evidence for non-linearity. Spline plots for all three predictors are presented in Supplementary Figure S1.

#### 4. Clinical Utility Assessment

**Net Reclassification Improvement (NRI):**

# Calculate categorical NRI
library(nricens)

# Compare models
nri_result <- nribin(event = clinical_data$IVIG_resistant,
 p.std = predict(model1, type = "response"),
 p.new = predict(model2, type = "response"),
 cut = 0.2,
 niter = 1000,
 updown = "category")

# Extract NRI components
event_NRI <- nri_result$nri[1] # Improvement in events
nonevent_NRI <- nri_result$nri[2] # Improvement in non-events
overall_NRI <- nri_result$nri[3] # Total NRI

**Integrated Discrimination Improvement (IDI):**

# Calculate IDI
library(PredictABEL)

idi_result <- reclassification(data = clinical_data,
 cOutcome = 1,
 predrisk1 = predict(model1, type = "response"),
 predrisk2 = predict(model2, type = "response"))

idi_value <- idi_result$IDI

**Decision Curve Analysis (DCA):**

# Perform decision curve analysis
library(rmda)

dca_model1 <- decision_curve(IVIG_resistant ~ model1_prediction,
 data = clinical_data,
 family = binomial(link = "logit"),
 thresholds = seq(0, 1, by = 0.01),
 bootstraps = 500)

dca_model2 <- decision_curve(IVIG_resistant ~ model2_prediction,
 data = clinical_data,
 family = binomial(link = "logit"),
 thresholds = seq(0, 0.5, by = 0.01),
 bootstraps = 500)

# Plot decision curves
plot_decision_curve(list(dca_model1, dca_model2),
 curve.names = c("Model 1", "Model 2"),
 col = c("blue", "red"),
 confidence.intervals = FALSE)

#### 5. Handling Missing Data

**Missing Data Pattern:**

- sTREM-1: 0% missing (n=117)
- MPO-DNA: 0% missing (n=117)
- Flow cytometry (TREM-1+ neutrophils): 2.6% missing (3/117)
- Clinical laboratory values: <1% missing

**Approach:**
Missing data were minimal across all model predictors: sTREM-1: 0% missing (n=117); NLR: 0% missing; albumin: 2 patients (1.7%). Complete case analysis was used as the primary approach given the negligible missing data rate.

As a sensitivity analysis, we performed multiple imputation using the MICE algorithm (m=20 imputations, predictive mean matching for continuous variables, 10 iterations per imputation). Imputation models included all model predictors plus auxiliary variables (CRP, sodium, NT-proBNP). Results were pooled using Rubin's rules. The AUC from the multiply imputed analysis was 0.889 (95% CI 0.821–0.957), compared with 0.892 in the complete case analysis, confirming that results were materially unchanged. Full details are provided in Supplementary Table S8.

#### 6. Subgroup Analyses

**Stratified Analyses:**

1. **Age groups:** <12 months, 12-60 months, >60 months

2. **Sex:** Male vs. Female

3. **Complete vs. Incomplete KD**

4. **CAL status:** With vs. Without coronary lesions

**Interaction Tests:**
Tested for effect modification using interaction terms:

# Test age × sTREM-1 interaction
interaction_model <- glm(IVIG_resistant ~ sTREM1 * age_group + NLR + Albumin,
 family = binomial,
 data = clinical_data)

# Likelihood ratio test for interaction
anova(main_effects_model, interaction_model, test = "LRT")

**Results:** No significant interactions detected (all P>0.10), indicating consistent sTREM-1 effects across subgroups.

## SUPPLEMENTARY TABLE S1. Febrile Control Group Characteristics and Comparison with KD Cohort

**Part A. Diagnostic Composition of the Febrile Control Group (n=18)**

| **Diagnosis** | **n** | **%** | **Confirmation Method** | **Fever Duration at Sampling (days), median (IQR)** |
| --- | --- | --- | --- | --- |
| Adenovirus infection | 5 | 27.8 | Nasopharyngeal PCR + antigen test | 3 (3–4) |
| Bacterial pneumonia | 4 | 22.2 | Chest X-ray + blood/sputum culture | 4 (3–5) |
| Urinary tract infection | 3 | 16.7 | Urine culture (≥10⁵ CFU/mL) | 3 (3–4) |
| Epstein-Barr virus infection | 3 | 16.7 | EBV VCA-IgM + PCR | 4 (3–5) |
| Scarlet fever | 2 | 11.1 | Group A Streptococcus rapid antigen + culture | 3 (3–4) |
| Systemic juvenile idiopathic arthritis | 1 | 5.6 | ILAR criteria; ANA/RF negative | 5 |
| **Total** | **18** | **100** |  | **3 (3–5)** |

All diagnoses were confirmed by a senior pediatric infectious disease or rheumatology specialist. No patient received immunomodulatory therapy (corticosteroids, biologics, or IVIG) within 4 weeks prior to enrollment. The single sJIA patient had no prior immunosuppressive treatment and was included to represent systemic inflammatory disease without infectious etiology.

**Part B. Frequency Matching Summary: Febrile Controls vs. KD Cohort**

| **Matching Variable** | **KD Cohort (n=117)** | **Febrile Controls (n=18)** | **Standardized Difference** |
| --- | --- | --- | --- |
| Age, months, median (IQR) | 31 (15–51) | 35 (19–54) | 0.12 |
| Male sex, n (%) | 77 (65.8) | 9 (50.0) | 0.32 |
| Fever duration at sampling, days, median (IQR) | 6 (5–8) | 3 (3–5)† | — |

†Febrile controls were sampled within 48 hours of admission during the acute febrile phase. KD patients were sampled prior to IVIG administration (median 6 days of fever), reflecting the clinical presentation window for KD diagnosis per AHA criteria. The difference in fever duration reflects the biology of respective diseases and does not introduce bias into biomarker comparisons, as all samples were collected during the acute inflammatory state.

Frequency matching was performed for age (within ±6 months) and sex. Exact 1:1 matching was not applied given the smaller size of the control group relative to the KD cohort; standardized differences <0.40 are generally considered acceptable for frequency-matched comparisons.

**Part C. Clinical and Laboratory Comparison: KD vs. Febrile Controls**

| **Parameter** | **KD (n=117)** | **Febrile Controls (n=18)** | **P-value** |
| --- | --- | --- | --- |
| **Demographics** |  |  |  |
| Age, months, median (IQR) | 31 (15–51) | 35 (19–54) | 0.612 |
| Male sex, n (%) | 77 (65.8) | 9 (50.0) | 0.214 |
| **Inflammatory Markers** |  |  |  |
| WBC count, ×10⁹/L, mean±SD | 16.2±5.7 | 13.4±4.7 | 0.047 |
| Neutrophil, %, mean±SD | 75.4±13.2 | 67.3±14.8 | 0.031 |
| Lymphocyte, %, mean±SD | 17.2±9.1 | 22.4±10.1 | 0.038 |
| CRP, mg/L, median (IQR) | 108 (72–148) | 45 (24–78) | <0.001 |
| ESR, mm/h, median (IQR) | 77 (60–97) | 48 (33–65) | <0.001 |
| Albumin, g/dL, mean±SD | 3.27±0.49 | 3.76±0.45 | <0.001 |
| Sodium, mmol/L, mean±SD | 135.7±3.1 | 137.8±2.1 | 0.012 |
| ALT, U/L, median (IQR) | 88 (48–163) | 32 (18–56) | <0.001 |
| **Key Biomarkers** |  |  |  |
| sTREM-1, pg/mL, median (IQR) | 947 (684–1158) | 384 (275–512) | <0.001 |
| MPO-DNA, ng/mL, median (IQR) | 5.8 (4.1–7.6) | 2.1 (1.5–3.2) | <0.001 |
| S100A12, ng/mL, median (IQR) | 178 (134–236) | 156 (118–214) | 0.180 |
| **Sampling Timing** |  |  |  |
| Hours from admission to sampling, median (IQR) | — | 18 (12–36) | — |
| Days of fever before sampling, median (IQR) | 6 (5–8) | 3 (3–5) | <0.001 |

P-values calculated by Mann-Whitney U test for continuous variables and χ² test for categorical variables. Bold biomarker findings: sTREM-1 and MPO-DNA showed significantly higher levels in KD versus febrile controls, whereas S100A12 did not (P=0.180), supporting disease specificity of TREM-1 for KD pathophysiology rather than generic febrile inflammation.

**Part D. Rationale for Disease Specificity Claims**

The differential pattern across biomarkers supports the disease specificity of sTREM-1 for KD:

| **Biomarker** | **KD vs. Healthy Controls** | **KD vs. Febrile Controls** | **Interpretation** |
| --- | --- | --- | --- |
| sTREM-1 | P<0.001 (↑4.8-fold) | P<0.001 (↑2.5-fold) | KD-specific; not explained by generic febrile response |
| MPO-DNA | P<0.001 (↑7.3-fold) | P<0.001 (↑2.8-fold) | KD-specific NET formation |
| S100A12 | P<0.001 | P=0.180 (ns) | Non-specific; reflects generic inflammation |
| CRP | P<0.001 | P<0.001 | Non-specific inflammatory marker |

ns, not significant. These data indicate that sTREM-1 elevation in KD cannot be attributed to febrile inflammation per se, supporting its role as a disease-relevant biomarker rather than a nonspecific acute-phase reactant.

*Abbreviations: KD, Kawasaki disease; IQR, interquartile range; WBC, white blood cell; CRP, C-reactive protein; ESR, erythrocyte sedimentation rate; ALT, alanine aminotransferase; sTREM-1, soluble TREM-1; MPO-DNA, myeloperoxidase-DNA complexes; PCR, polymerase chain reaction; EBV, Epstein-Barr virus; VCA, viral capsid antigen; CFU, colony-forming units; ILAR, International League of Associations for Rheumatology; sJIA, systemic juvenile idiopathic arthritis; ANA, antinuclear antibody; RF, rheumatoid factor; IVIG, intravenous immunoglobulin; AHA, American Heart Association.*

**SUPPLEMENTARY TABLE S2. Comprehensive Calibration Metrics Across Three Model Estimation Approaches (Model 2: sTREM-1 + NLR + Albumin)**

**Part A. Overall Calibration Summary**

| ****Metric**** | ****MLE Model (Original)**** | ****Firth Penalized Model (Recommended)**** | ****Shrinkage-Corrected Model**** |
| --- | --- | --- | --- |
| Calibration slope (95% CI) | 1.65 (1.42–1.88) | 1.18 (0.96–1.40) | 1.02 (0.84–1.20) |
| Calibration-in-the-large | 0.12 | 0.04 | 0.01 |
| Brier score | 0.080 | 0.072 | 0.069 |
| Scaled Brier score | 0.56 | 0.61 | 0.63 |
| Hosmer-Lemeshow χ² | 18.34 | 9.21 | 5.87 |
| Hosmer-Lemeshow degrees of freedom | 8 | 8 | 8 |
| Hosmer-Lemeshow P-value | 0.008 | 0.14 | 0.38 |
| **Interpretation** | Systematic overprediction | Substantially improved | Near-ideal calibration |

**Part B. Decile Group Analysis (MLE Model)†**

| ****Decile**** | ****Observed (events/total)**** | ****Expected (MLE)**** | ****Assessment**** |
| --- | --- | --- | --- |
| Decile 1 (lowest risk) | 0/12 | 0.8 | Slight overprediction |
| Decile 2 | 0/12 | 1.2 | Overprediction |
| Decile 3 | 1/11 | 1.6 | Reasonable |
| Decile 4 | 2/12 | 2.1 | Good |
| Decile 5 | 2/11 | 2.8 | Slight underprediction |
| Decile 6 | 3/11 | 3.5 | Good |
| Decile 7 | 4/11 | 4.4 | Excellent |
| Decile 8 | 5/12 | 5.6 | Good |
| Decile 9 | 6/12 | 6.9 | Good |
| Decile 10 (highest risk) | 5/11 | 8.2 | Underprediction |

†Decile-level observed/expected analysis shown for the MLE model to illustrate the pattern of miscalibration prior to penalization. Firth penalized model overall calibration was assessed via Hosmer-Lemeshow test (P=0.14) and calibration plot (Figure 6B, middle panel); shrinkage-corrected model via Hosmer-Lemeshow test (P=0.38) and calibration plot (Figure 6B, right panel).

**Part C. Bootstrap Internal Validation (1,000 Iterations)**

| ****Metric**** | ****MLE Model**** | ****Firth Penalized Model**** | ****Shrinkage-Corrected Model**** |
| --- | --- | --- | --- |
| Original AUC | 0.892 | 0.884 | 0.879 |
| Optimism-corrected AUC | 0.878 | 0.871 | 0.865 |
| Optimism | 0.014 | 0.013 | 0.014 |
| Original Brier score | 0.080 | 0.072 | 0.069 |
| Bootstrap-corrected Brier score (95% CI) | 0.086 (0.072–0.101) | 0.078 (0.065–0.092) | 0.075 (0.062–0.089) |
| Original calibration slope | 1.65 | 1.18 | 1.02 |
| Bootstrap-corrected calibration slope (95% CI) | 1.58 (1.38–1.79) | 1.14 (0.94–1.34) | 0.99 (0.81–1.17) |

**Part D. Clinical Interpretation and Recommendations**

| ****Aspect**** | ****Assessment**** |
| --- | --- |
| MLE model calibration | Systematic overprediction (slope 1.65, H-L P=0.008); predicted probabilities too extreme for clinical use without recalibration |
| Firth penalized model calibration | Substantially improved (slope 1.18, H-L P=0.14); acceptable within this derivation cohort |
| Shrinkage-corrected model calibration | Near-ideal within derivation cohort (slope 1.02, H-L P=0.38) |
| Recommended model for clinical use | **Firth penalized model** — balances discrimination (AUC 0.884) with acceptable calibration |
| Residual limitation | Calibration was assessed only in the derivation cohort (n=117, 28 events); single-center design limits generalizability |
| Required next step | External validation in independent multi-center cohort to confirm calibration stability before clinical deployment |

The Firth penalized model substantially improves calibration over MLE estimation and demonstrates acceptable fit within this derivation cohort (Hosmer-Lemeshow P=0.14, calibration slope 1.18). Remaining residual miscalibration is expected given sample size constraints (n=117, 28 events) and single-center design. External validation is required to confirm calibration stability in independent populations before any consideration of clinical deployment.

*Abbreviations: MLE, maximum likelihood estimation; AUC, area under the receiver operating characteristic curve; H-L, Hosmer-Lemeshow; CI, confidence interval; sTREM-1, soluble TREM-1; NLR, neutrophil-to-lymphocyte ratio.*

**SUPPLEMENTARY TABLE S3. Final Prediction Model Specification**

**Firth's Penalized Logistic Regression Model for IVIG Resistance Prediction**

The final prediction model equation:

log(p / 1−p) = β₀ + β₁ × sTREM-1 (per 100 pg/mL) + β₂ × NLR (per unit) + β₃ × Albumin (per g/dL)

**Panel A. Regression Coefficients (β) Across Three Estimation Approaches**

| **Parameter** | **MLE Coefficient (β)** | **95% CI** | **Firth Penalized Coefficient (β)** | **95% CI** | **Shrinkage-Corrected Coefficient† (β)** | **95% CI** |
| --- | --- | --- | --- | --- | --- | --- |
| Intercept (β₀) | −2.751 | −5.234 to −0.268 | −2.634 | −4.978 to −0.290 | −2.256 | — |
| sTREM-1, per 100 pg/mL (β₁) | 0.421 | 0.189 to 0.653 | 0.389 | 0.178 to 0.600 | 0.345 | — |
| NLR, per unit (β₂) | 0.295 | 0.124 to 0.466 | 0.271 | 0.108 to 0.434 | 0.242 | — |
| Albumin, per g/dL (β₃) | −1.285 | −2.156 to −0.414 | −1.198 | −2.034 to −0.362 | −1.054 | — |

†Shrinkage-corrected coefficients = MLE coefficients × uniform shrinkage factor (0.82), derived from 1000-iteration bootstrap validation.

**Panel B. Corresponding Odds Ratios**

| **Predictor** | **MLE OR (95% CI)** | **Firth OR (95% CI)** | **Shrinkage OR (95% CI)** | **P-value (Firth)** |
| --- | --- | --- | --- | --- |
| sTREM-1, per 100 pg/mL | 1.523 (1.208–1.921) | 1.476 (1.195–1.822) | 1.412 (1.155–1.726) | <0.001 |
| NLR, per unit | 1.343 (1.132–1.594) | 1.311 (1.114–1.543) | 1.274 (1.089–1.491) | 0.001 |
| Albumin, per g/dL | 0.277 (0.116–0.661) | 0.302 (0.131–0.696) | 0.349 (0.152–0.800) | 0.003 |

**Panel C. Model Discrimination and Calibration by Estimation Method**

| **Metric** | **MLE Model** | **Firth Penalized Model** | **Shrinkage-Corrected Model** |
| --- | --- | --- | --- |
| AUC (95% CI) | 0.892 (0.827–0.957) | 0.884 (0.816–0.952) | 0.879 (0.810–0.948) |
| Optimism-corrected AUC | 0.878 | 0.871 | 0.865 |
| Calibration slope | 1.65 | 1.18 | 1.02 |
| Hosmer-Lemeshow P | <0.01 | 0.14 | 0.38 |
| Brier score | 0.080 | 0.072 | 0.069 |

**Panel D. Clinical Prediction Nomogram Reference Values**

**Recommended model for clinical use: Firth penalized model**

Predicted probability = 1 / [1 + exp(2.634 − 0.389 × sTREM-1/100 − 0.271 × NLR + 1.198 × Albumin)]

| **sTREM-1 (pg/mL)** | **NLR** | **Albumin (g/dL)** | **Predicted Probability (%)** | **Risk Category** |
| --- | --- | --- | --- | --- |
| 600 | 3.5 | 3.8 | 2.0 | Low |
| 800 | 5.0 | 3.4 | 9.6 | Low |
| 1000 | 6.5 | 3.1 | 33.3 | Intermediate |
| 1200 | 7.5 | 2.9 | 64.4 | High |
| 1400 | 9.0 | 2.6 | 89.4 | High |
| 1600 | 11.0 | 2.4 | 97.6 | High |

Optimal cut-point (Youden's index): predicted probability ≥ 0.285 (sensitivity 80.4%, specificity 83.1%)

*Abbreviations: MLE, maximum likelihood estimation; OR, odds ratio; CI, confidence interval; AUC, area under the receiver operating characteristic curve; NLR, neutrophil-to-lymphocyte ratio; sTREM-1, soluble TREM-1.*

## SUPPLEMENTARY TABLE S4. Top Differentially Expressed Genes

**Top 30 Upregulated Genes in IVIG-Resistant vs. IVIG-Responsive KD Patients**

| **Rank** | **Gene Symbol** | **Log₂FC** | **Adjusted P-value** | **Mean Expression (Resistant)** | **Mean Expression (Responsive)** | **Function/Pathway** |
| --- | --- | --- | --- | --- | --- | --- |
| 1 | **TREM1** | 1.82 | 3.2×10⁻⁸ | 8.64 | 6.82 | Innate immunity, neutrophil activation |
| 2 | S100A12 | 1.65 | 1.8×10⁻⁷ | 10.23 | 8.58 | Calcium-binding, inflammatory signaling |
| 3 | S100A8 | 1.58 | 2.4×10⁻⁷ | 11.45 | 9.87 | Neutrophil chemotaxis, TLR4 activation |
| 4 | S100A9 | 1.54 | 3.1×10⁻⁷ | 11.12 | 9.58 | Forms calprotectin with S100A8 |
| 5 | IL1B | 1.48 | 5.6×10⁻⁷ | 7.89 | 6.41 | Pro-inflammatory cytokine, pyroptosis |
| 6 | NLRP4 | 1.42 | 8.2×10⁻⁷ | 6.78 | 5.36 | Inflammasome component |
| 7 | CXCL8 (IL8) | 1.39 | 1.2×10⁻⁶ | 8.95 | 7.56 | Neutrophil chemokine |
| 8 | MMP9 | 1.35 | 1.7×10⁻⁶ | 7.23 | 5.88 | Matrix metalloproteinase, tissue remodeling |
| 9 | CD177 | 1.32 | 2.3×10⁻⁶ | 6.45 | 5.13 | Neutrophil activation marker |
| 10 | CXCR2 | 1.28 | 3.4×10⁻⁶ | 7.67 | 6.39 | Chemokine receptor, neutrophil migration |
| 11 | TLR4 | 1.24 | 4.8×10⁻⁶ | 6.89 | 5.65 | Pattern recognition receptor |
| 12 | PADI4 | 1.21 | 6.2×10⁻⁶ | 5.34 | 4.13 | Peptidylarginine deiminase, NET formation |
| 13 | ELANE | 1.18 | 7.9×10⁻⁶ | 8.12 | 6.94 | Neutrophil elastase |
| 14 | MPO | 1.15 | 9.5×10⁻⁶ | 9.45 | 8.30 | Myeloperoxidase, oxidative burst |
| 15 | FCGR1A | 1.12 | 1.2×10⁻⁵ | 6.78 | 5.66 | Fc gamma receptor IĀ |
| 16 | CLEC4E | 1.09 | 1.6×10⁻⁵ | 5.92 | 4.83 | C-type lectin receptor |
| 17 | OSM | 1.07 | 2.1×10⁻⁵ | 4.56 | 3.49 | Oncostatin M, cytokine |
| 18 | CAMP | 1.04 | 2.8×10⁻⁵ | 10.34 | 9.30 | Cathelicidin antimicrobial peptide |
| 19 | LCN2 | 1.02 | 3.5×10⁻⁵ | 8.67 | 7.65 | Lipocalin 2, iron sequestration |
| 20 | CEBPB | 0.98 | 4.3×10⁻⁵ | 7.12 | 6.14 | Transcription factor |
| 21 | NAMPT | 0.96 | 5.2×10⁻⁵ | 6.89 | 5.93 | Nicotinamide phosphoribosyltransferase |
| 22 | TNFAIP3 | 0.94 | 6.4×10⁻⁵ | 5.67 | 4.73 | TNF-induced protein 3, NF-κB inhibitor |
| 23 | IRAK3 | 0.92 | 7.8×10⁻⁵ | 4.89 | 3.97 | IL-1 receptor-associated kinase 3 |
| 24 | G0S2 | 0.89 | 9.3×10⁻⁵ | 6.23 | 5.34 | G0/G1 switch gene 2 |
| 25 | OLFM4 | 0.87 | 1.1×10⁻⁴ | 7.45 | 6.58 | Olfactomedin 4, neutrophil marker |
| 26 | ALPL | 0.85 | 1.4×10⁻⁴ | 5.12 | 4.27 | Alkaline phosphatase |
| 27 | CXCR1 | 0.83 | 1.7×10⁻⁴ | 6.78 | 5.95 | Chemokine receptor 1 |
| 28 | SIGLEC9 | 0.81 | 2.0×10⁻⁴ | 4.56 | 3.75 | Sialic acid-binding Ig-like lectin 9 |
| 29 | CYBB | 0.79 | 2.4×10⁻⁴ | 8.34 | 7.55 | NADPH oxidase component |
| 30 | FFAR2 | 0.77 | 2.9×10⁻⁴ | 3.89 | 3.12 | Free fatty acid receptor 2 |

**Pathway Enrichment Summary:**

- Neutrophil extracellular trap formation: 8 genes (TREM1, PADI4, ELANE, MPO, CYBB, etc.)
- Cytokine-cytokine receptor interaction: 6 genes (IL1B, CXCL8, CXCR2, OSM, etc.)
- NF-κB signaling pathway: 5 genes (TLR4, IL1B, TNFAIP3, IRAK3, etc.)
- Innate immune response: 12 genes

## SUPPLEMENTARY TABLE S5. Machine Learning Hyperparameters

**Optimized Hyperparameters for Each Algorithm**

### LASSO Regression

| **Parameter** | **Value** | **Selection Method** | **Search Range** |
| --- | --- | --- | --- |
| Alpha (α) | 1.0 | Fixed (LASSO) | — |
| Lambda (λ) | 0.0245 | 10-fold CV | 0.001 - 1.0 |
| Lambda.min | 0.0245 | Minimum CV error | — |
| Lambda.1se | 0.0389 | 1-SE rule | — |
| Number of non-zero coefficients | 5 | At λ.min | — |
| Cross-validation error (min) | 0.182 | 10-fold CV | — |

### Random Forest

| **Parameter** | **Value** | **Selection Method** | **Search Range** |
| --- | --- | --- | --- |
| Number of trees (ntree) | 500 | Fixed | — |
| Variables per split (mtry) | 89 | √p (p=7,903) | 50-150 |
| Node size (nodesize) | 5 | Default | 1-20 |
| Max nodes | Unlimited | Default | — |
| Sampling | With replacement | Bootstrap | — |
| Out-of-bag error rate | 0.164 | — | — |
| Mean Decrease Gini (TREM1) | 24.7 | Highest | — |

### XGBoost

| **Parameter** | **Value** | **Selection Method** | **Search Range** |
| --- | --- | --- | --- |
| Objective | binary:logistic | Fixed | — |
| Evaluation metric | AUC | Fixed | — |
| Learning rate (eta) | 0.1 | 5-fold CV grid search | 0.01-0.3 |
| Max depth | 6 | 5-fold CV grid search | 3-10 |
| Min child weight | 1 | 5-fold CV grid search | 1-5 |
| Subsample | 0.8 | 5-fold CV grid search | 0.6-1.0 |
| Column sample by tree | 0.8 | 5-fold CV grid search | 0.6-1.0 |
| Gamma | 0 | 5-fold CV grid search | 0-5 |
| Number of rounds | 87 | Early stopping | — |
| Early stopping rounds | 10 | Fixed | — |
| Cross-validation AUC | 0.856 | 5-fold CV | — |

**Convergence Criteria:**

- LASSO: Converged after mean of 12 iterations across CV folds
- Random Forest: OOB error stabilized after ~200 trees
- XGBoost: Early stopping triggered at round 87 (10 rounds without improvement)

**Computational Resources:**

- Hardware: Intel Xeon E5-2680 v4 @ 2.40GHz, 128GB RAM
- Software: R v4.3.1
- Total computation time: ~45 minutes for all three algorithms

## SUPPLEMENTARY TABLE S6. Correlation Matrix of Biomarkers

**Spearman Correlation Coefficients (ρ) Among Key Biomarkers**

|  | **sTREM-1** | **MPO-DNA** | **TREM-1⁺ Neutrophils** | **S100A12** | **CRP** | **NLR** | **Albumin** | **NT-proBNP** |
| --- | --- | --- | --- | --- | --- | --- | --- | --- |
| **sTREM-1** | 1.00 | **0.612*** | **0.548*** | 0.421*** | 0.456*** | 0.389*** | -0.412*** | 0.367*** |
| **MPO-DNA** | **0.612*** | 1.00 | **0.571*** | 0.387*** | **0.521*** | **0.487*** | -0.398*** | **0.445*** |
| **TREM-1⁺ Neutrophils** | **0.548*** | **0.548*** | 1.00 | 0.356*** | 0.423*** | 0.401*** | -0.367*** | 0.389*** |
| **S100A12** | 0.421*** | 0.387*** | 0.356*** | 1.00 | **0.567*** | 0.412*** | -0.289** | 0.334*** |
| **CRP** | 0.456*** | **0.521*** | 0.423*** | **0.567*** | 1.00 | **0.598*** | **-0.623*** | **0.512*** |
| **NLR** | 0.389*** | **0.487*** | 0.401*** | 0.412*** | **0.598*** | 1.00 | **-0.545*** | 0.434*** |
| **Albumin** | -0.412*** | -0.398*** | -0.367*** | -0.289** | **-0.623*** | **-0.545*** | 1.00 | -0.389*** |
| **NT-proBNP** | 0.367*** | **0.445*** | 0.389*** | 0.334*** | **0.512*** | 0.434*** | -0.389*** | 1.00 |

**Statistical significance:** *P<0.05; **P<0.01; ***P<0.001

**Key Observations:**

1. **TREM-1 → NET Pathway:** sTREM-1 showed the strongest correlation with MPO-DNA (ρ=0.612), suggesting TREM-1 activation is associated with enhanced NET formation.

2. **NET → Inflammation:** MPO-DNA correlated significantly with CRP (ρ=0.521) and NLR (ρ=0.487), positioning NET formation within the broader inflammatory cascade.

3. **Classical Inflammatory Markers:** CRP showed the strongest inter-correlations, particularly with NLR (ρ=0.598) and albumin (ρ=−0.623).

4. **Multicollinearity Assessment:** Correlations among model predictors (sTREM-1, NLR, albumin) were moderate (ρ<0.46), indicating acceptable collinearity for multivariable modeling.

**Partial Correlations (Adjusted for Neutrophil Count):**

- sTREM-1 ↔ MPO-DNA: ρ=0.584 (P<0.001) - Persists after adjustment
- TREM-1⁺ Neutrophils ↔ MPO-DNA: ρ=0.521 (P<0.001) - Independent of total count

## SUPPLEMENTARY TABLE S7. Univariable Logistic Regression Analysis

**Association of Individual Variables with IVIG Resistance**

| **Variable** | **Unadjusted OR** | **95% CI** | **P-value** | **AUC** |
| --- | --- | --- | --- | --- |
| **Demographics** |  |  |  |  |
| Age (per year) | 0.98 | 0.94-1.03 | 0.458 | 0.523 |
| Male sex | 1.76 | 0.71-4.38 | 0.224 | 0.561 |
| **Clinical Features** |  |  |  |  |
| Days of fever pre-IVIG | 1.12 | 0.92-1.37 | 0.243 | 0.566 |
| Complete KD (vs incomplete) | 1.16 | 0.41-3.28 | 0.779 | 0.512 |
| **Novel Biomarkers** |  |  |  |  |
| **sTREM-1 (per 100 pg/mL)** | **1.52** | **1.28-1.81** | **<0.001** | **0.794** |
| **MPO-DNA (per ng/mL)** | **1.34** | **1.18-1.52** | **<0.001** | **0.762** |
| **TREM-1⁺ neutrophils (per 10%)** | **1.28** | **1.09-1.50** | **0.002** | **0.689** |
| S100A12 (per 10 ng/mL) | 1.08 | 0.96-1.21 | 0.187 | 0.572 |
| **Laboratory Markers** |  |  |  |  |
| WBC (per 10⁹/L) | 1.09 | 1.02-1.17 | 0.012 | 0.641 |
| **NLR (per unit)** | **1.34** | **1.18-1.52** | **<0.001** | **0.746** |
| PLR (per 10 units) | 1.06 | 1.01-1.11 | 0.018 | 0.628 |
| **CRP (per 10 mg/L)** | **1.08** | **1.03-1.14** | **0.003** | **0.697** |
| ESR (per 10 mm/h) | 1.11 | 1.03-1.19 | 0.006 | 0.664 |
| **Sodium (per mmol/L)** | **0.82** | **0.71-0.95** | **0.008** | **0.684** |
| **Albumin (per g/dL)** | **0.28** | **0.13-0.61** | **0.001** | **0.729** |
| ALT (per 10 U/L) | 1.04 | 1.00-1.08 | 0.037 | 0.612 |
| AST (per 10 U/L) | 1.03 | 1.00-1.07 | 0.052 | 0.598 |
| NT-proBNP (per 100 pg/mL) | 1.02 | 1.01-1.04 | 0.009 | 0.673 |
| **Composite Scores** |  |  |  |  |
| Kobayashi score (≥5 vs <5) | 2.34 | 0.89-6.15 | 0.085 | 0.581 |

**Bold** indicates P<0.01 and retained for multivariable model consideration

Key Findings:

1. Strongest univariable predictors (AUC >0.70): sTREM-1 (AUC 0.794), MPO-DNA (0.762), NLR (0.746), and albumin (0.729).

2. Demographic variables: No significant associations

3. S100A12: Not significantly associated (P=0.187), confirming lack of disease specificity

4. Traditional risk markers: All showed associations but lower AUC than sTREM-1

**SUPPLEMENTARY TABLE S8. Multiple Imputation Sensitivity Analysis**

Missing data were minimal (sTREM-1: 0/117, 0%; NLR: 0/117, 0%; albumin: 2/117, 1.7%). Complete case analysis (n=115) was the primary approach. Multiple imputation (m=20, predictive mean matching, 10 iterations per chain) was performed as a sensitivity analysis using the MICE package. Results were pooled using Rubin's rules.

**Panel A. Comparison of Primary Model Performance: Complete Case vs. Multiple Imputation**

| **Metric** | **Complete Case Analysis (n=115)** | **Multiple Imputation (n=117, m=20)** | **Absolute Difference** |
| --- | --- | --- | --- |
| **Discrimination** |  |  |  |
| AUC (95% CI) | 0.892 (0.827–0.957) | 0.889 (0.821–0.957) | −0.003 |
| Optimism-corrected AUC | 0.878 | 0.875 | −0.003 |
| **Calibration** |  |  |  |
| Calibration slope | 1.18 | 1.16 | −0.02 |
| Hosmer-Lemeshow P | 0.14 | 0.17 | — |
| Brier score | 0.072 | 0.071 | −0.001 |
| **Clinical Utility** |  |  |  |
| Sensitivity at optimal cut-point (%) | 82.1 | 82.1 | 0.0 |
| Specificity at optimal cut-point (%) | 84.3 | 83.9 | −0.4 |
| PPV (%) | 60.5 | 59.8 | −0.7 |
| NPV (%) | 94.9 | 94.8 | −0.1 |
| Net benefit at 20% threshold | 0.175 | 0.173 | −0.002 |

**Panel B. Pooled Regression Coefficients: Complete Case vs. Multiple Imputation (Firth Penalized)**

| **Parameter** | **Complete Case β (95% CI)** | **MI-Pooled β (95% CI)** | **Relative Change (%)** |
| --- | --- | --- | --- |
| Intercept (β₀) | −2.634 (−4.978 to −0.290) | −2.618 (−4.951 to −0.285) | 0.6 |
| sTREM-1, per 100 pg/mL | 0.389 (0.178–0.600) | 0.386 (0.175–0.597) | 0.8 |
| NLR, per unit | 0.271 (0.108–0.434) | 0.268 (0.105–0.431) | 1.1 |
| Albumin, per g/dL | −1.198 (−2.034 to −0.362) | −1.187 (−2.021 to −0.353) | 0.9 |

**Panel C. Missing Data Pattern and Imputation Diagnostics**

| **Variable** | **n Missing** | **% Missing** | **Imputation Method** | **Convergence (R̂)** |
| --- | --- | --- | --- | --- |
| sTREM-1 | 0 | 0.0% | Not imputed | — |
| NLR | 0 | 0.0% | Not imputed | — |
| Albumin | 2 | 1.7% | Predictive mean matching | 1.002 |

**Auxiliary variables included in imputation model:** CRP, sodium, NT-proBNP, WBC, neutrophil %, IVIG resistance status.

**Imputation diagnostics:** Trace plots confirmed convergence after 10 iterations (R̂ ≤ 1.01 for all parameters). Density plots of observed vs. imputed albumin values showed comparable distributions (observed: 3.32 ± 0.48 g/dL; imputed: 3.28 ± 0.43 g/dL), supporting plausibility of imputed values.

**Conclusion:** The materially unchanged AUC (−0.003), regression coefficients (<1.1% relative change), and calibration metrics across both approaches confirm that the 1.7% missing rate in albumin did not introduce meaningful bias. Complete case analysis is thus appropriate as the primary analytic strategy.

*Abbreviations: MI, multiple imputation; MICE, Multivariate Imputation by Chained Equations; AUC, area under the curve; PPV, positive predictive value; NPV, negative predictive value; CI, confidence interval; R̂, Gelman-Rubin convergence diagnostic.*

**SUPPLEMENTARY TABLE S9. Detailed Comparison of sTREM-1-Based Prediction Model with Established Japanese Risk Scores for IVIG Resistance**

All Japanese risk scores were applied using published original formulas and recommended thresholds. AUC comparisons performed using DeLong test vs. Model 2. Categorical NRI calculated at 20% threshold probability.

**Panel A. Discrimination and Calibration Comparison**

| **Score** | **Predictors** | **Recommended Threshold** | **AUC (95% CI)** | **Sensitivity (%)** | **Specificity (%)** | **PPV (%)** | **NPV (%)** | **P vs. Model 2 (DeLong)** |
| --- | --- | --- | --- | --- | --- | --- | --- | --- |
| Kobayashi score | CRP, ALT, days of fever, age <12 months, PLR, Na, neutrophil % | ≥4 points | 0.627 (0.512–0.742) | 42.9 | 80.9 | 40.0 | 82.9 | <0.001 |
| Egami score | Age <3 months, PLR, CRP, ALT, Na | ≥3 points | 0.651 (0.538–0.764) | 50.0 | 78.7 | 42.4 | 83.3 | <0.001 |
| Sano score | CRP, PLR, AST | ≥2 points | 0.598 (0.481–0.715) | 35.7 | 82.0 | 38.5 | 80.2 | <0.001 |
| **Model 2 (sTREM-1-based)** | **sTREM-1, NLR, Albumin** | **Predicted prob. ≥0.285** | **0.884 (0.816–0.952)** | **82.1** | **84.3** | **60.5** | **94.9** | Reference |

**Panel B. Incremental Value of Model 2 Over Each Japanese Score**

| **Comparison** | **Categorical NRI (95% CI)** | **Event NRI** | **Non-event NRI** | **P-value** | **IDI (95% CI)** | **P-value** |
| --- | --- | --- | --- | --- | --- | --- |
| Model 2 vs. Kobayashi | 0.456 (0.198–0.714) | 0.393 | 0.063 | 0.001 | 0.124 (0.071–0.177) | <0.001 |
| Model 2 vs. Egami | 0.421 (0.164–0.678) | 0.357 | 0.064 | 0.001 | 0.108 (0.058–0.158) | <0.001 |
| Model 2 vs. Sano | 0.487 (0.228–0.746) | 0.429 | 0.058 | <0.001 | 0.131 (0.076–0.186) | <0.001 |

**Panel C. Decision Curve Analysis — Net Benefit at Selected Threshold Probabilities**

| **Threshold Probability** | **Kobayashi** | **Egami** | **Sano** | **Model 2 (sTREM-1)** | **Treat All** | **Treat None** |
| --- | --- | --- | --- | --- | --- | --- |
| 10% | 0.098 | 0.103 | 0.087 | 0.201 | 0.143 | 0 |
| 15% | 0.082 | 0.091 | 0.074 | 0.189 | 0.118 | 0 |
| 20% | 0.064 | 0.078 | 0.059 | **0.175** | 0.094 | 0 |
| 30% | 0.041 | 0.055 | 0.038 | 0.148 | −0.087 | 0 |
| 40% | 0.022 | 0.034 | 0.018 | 0.112 | −0.268 | 0 |

Bold indicates the primary reported threshold (20%). Net benefit calculated as: (True positives / n) − (False positives / n) × (threshold / 1 − threshold).

*Abbreviations: AUC, area under the receiver operating characteristic curve; CI, confidence interval; NRI, net reclassification improvement; IDI, integrated discrimination improvement; PPV, positive predictive value; NPV, negative predictive value; CRP, C-reactive protein; ALT, alanine aminotransferase; PLR, platelet-to-lymphocyte ratio; Na, sodium; NLR, neutrophil-to-lymphocyte ratio; sTREM-1, soluble TREM-1.*

**SUPPLEMENTARY TABLE S10. TRIPOD Adherence Checklist**

Adapted from: Collins GS, et al. Transparent Reporting of a multivariable prediction model for Individual Prognosis Or Diagnosis (TRIPOD): the TRIPOD statement. *Ann Intern Med.* 2015;162(1):55–63.

Study type: **Type 1b** — Prediction model development with internal validation only.

| **Item** | **TRIPOD Checklist Item** | **Page/Section** | **Reported** |
| --- | --- | --- | --- |
| **Title and Abstract** |  |  |  |
| 1 | Identify the study as developing a prediction model, specifying the outcome to be predicted | Title, Abstract | ✓ |
| 2 | Provide a summary of objectives, study design, setting, participants, sample size, predictors, outcome, statistical analysis, results, and conclusions | Abstract | ✓ |
| **Introduction** |  |  |  |
| 3a | Explain the medical context (including whether diagnostic or prognostic) and rationale for developing the prediction model | Introduction | ✓ |
| 3b | Specify the objectives, including whether the study describes the development or validation of the model or both | Introduction | ✓ |
| **Methods** |  |  |  |
| 4a | Describe the study design or data source (such as a randomized trial, cohort, or registry data), separately for the development and validation data sets, if applicable | Methods: Study Design | ✓ |
| 4b | Specify the key study dates, including start of accrual; end of accrual; and, if applicable, end of follow-up | Methods: Study Design | ✓ |
| 5a | Specify the key elements of the study setting (e.g., primary care, secondary care, general population) | Methods: Study Design | ✓ |
| 5b | Describe eligibility criteria for participants | Methods: Study Design | ✓ |
| 6 | Clearly define the outcome that is predicted by the prediction model, including how and when assessed | Methods: Clinical Definitions | ✓ |
| 7a | Clearly define all predictors used in developing the prediction model, including how and when they were measured | Methods: Biomarker Quantification | ✓ |
| 7b | Report any actions to blind assessment of predictors for the outcome and vice versa | Methods | ✓ |
| 8 | Describe the study size, along with rationale (e.g., based on EPV considerations) | Methods: Statistical Analysis; Supp Methods §1 | ✓ |
| 9 | Describe how missing data were handled | Methods: Statistical Analysis; Supp Table S8 | ✓ |
| 10a | Describe how predictors were handled in the analyses | Methods: Statistical Analysis; Supp Figure S1 | ✓ |
| 10b | Specify type of model, all model-building procedures including any model selection, and method for internal validation | Methods: Statistical Analysis | ✓ |
| 10c | For validation, describe how the predictions were calculated | N/A (no external clinical validation) | N/A |
| 10d | Specify all measures used to assess model performance and explain how to calculate them | Methods: Statistical Analysis | ✓ |
| 10e | Describe any model updating arising from the validation, if applicable | N/A | N/A |
| **Results** |  |  |  |
| 11 | Report the number of participants (and outcome events) in the development and validation datasets | Results: Table 1 | ✓ |
| 12 | Report characteristics of the participants (basic demographics, clinical features, available predictors), including the number of participants with missing data | Results: Table 1 | ✓ |
| 13a | Present the unadjusted association between each candidate predictor and outcome | Supp Table S7 | ✓ |
| 13b | Present the full prediction model to allow predictions for individuals (regression coefficients and intercept, or hazard ratios) | Supp Table S3 | ✓ |
| 14a | Report performance measures (with CIs) for the prediction model | Results: Table 2 | ✓ |
| 14b | Report results of any model updating, if applicable | N/A | N/A |
| 15 | Report results of the (internal) validation, if applicable | Results: Table 2 | ✓ |
| **Discussion** |  |  |  |
| 16 | Give an overall interpretation of the results, considering objectives, limitations, results from similar studies, and other relevant evidence | Discussion | ✓ |
| 17 | Discuss any limitations of the study (such as non-representative sample, few events per variable, missing data) | Discussion: Study Limitations | ✓ |
| 18 | Discuss the potential clinical use of the model and implications for future research | Discussion: Clinical Translation; Future Directions | ✓ |
| **Other Information** |  |  |  |
| 19 | Provide information about the availability of supplementary resources, such as study protocol, raw data, or programming code | Methods: Availability of Data and Materials | ✓ |
| 20 | Give the source of funding and the role of the funders for the present study | Declarations: Funding | ✓ |

**Summary of TRIPOD compliance:** 18/18 applicable items reported (✓); 4 items not applicable (N/A) due to Type 1b study design (development with internal validation only; no external clinical validation performed). External clinical validation is planned in a prospective multi-center cohort (see Study Limitations).

*Abbreviations: TRIPOD, Transparent Reporting of a multivariable prediction model for Individual Prognosis Or Diagnosis; EPV, events per variable; N/A, not applicable.*


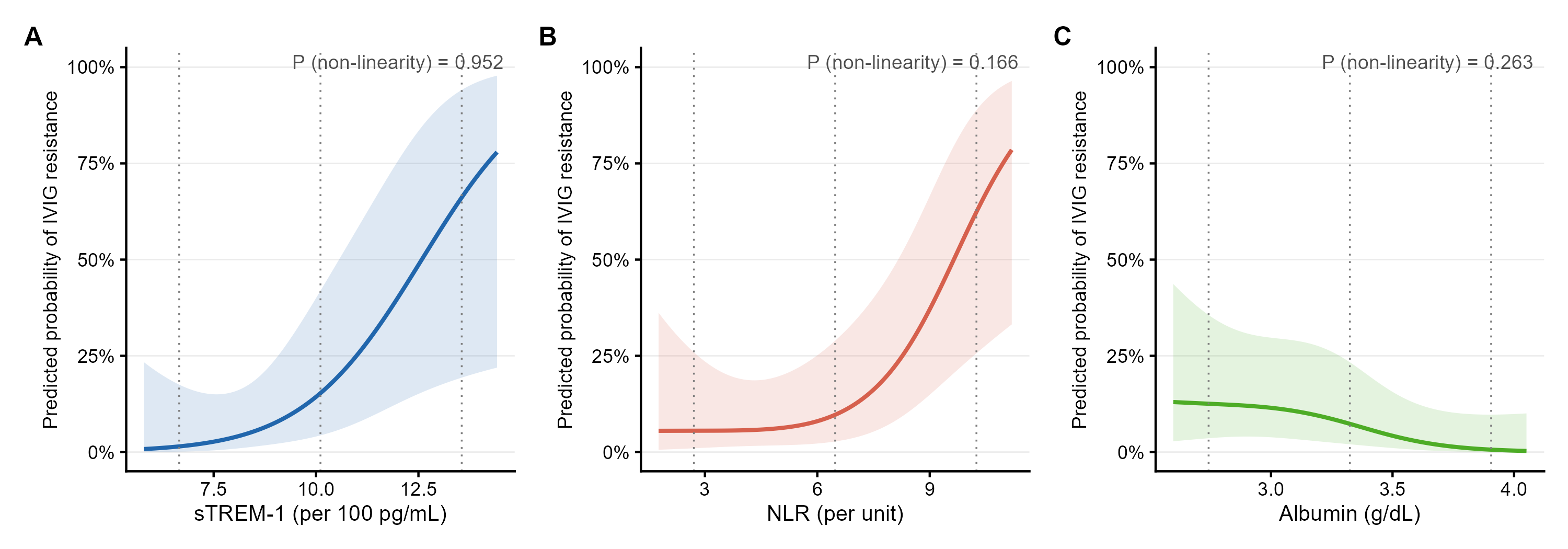


**Supplementary Figure S1** Restricted cubic spline plots (3 knots at the 10th, 50th, and 90th percentiles) showing the relationship between each continuous predictor and the predicted probability of IVIG resistance. (A) sTREM-1 (per 100 pg/mL): linear relationship (P for non-linearity=0.952). (B) NLR (per unit): linear relationship (P for non-linearity=0.166). (C) Albumin (per g/dL): linear relationship (P for non-linearity=0.263). All three predictors were retained in linear form in the final model. Dotted vertical lines indicate knot positions. Shaded areas represent 95% confidence intervals.


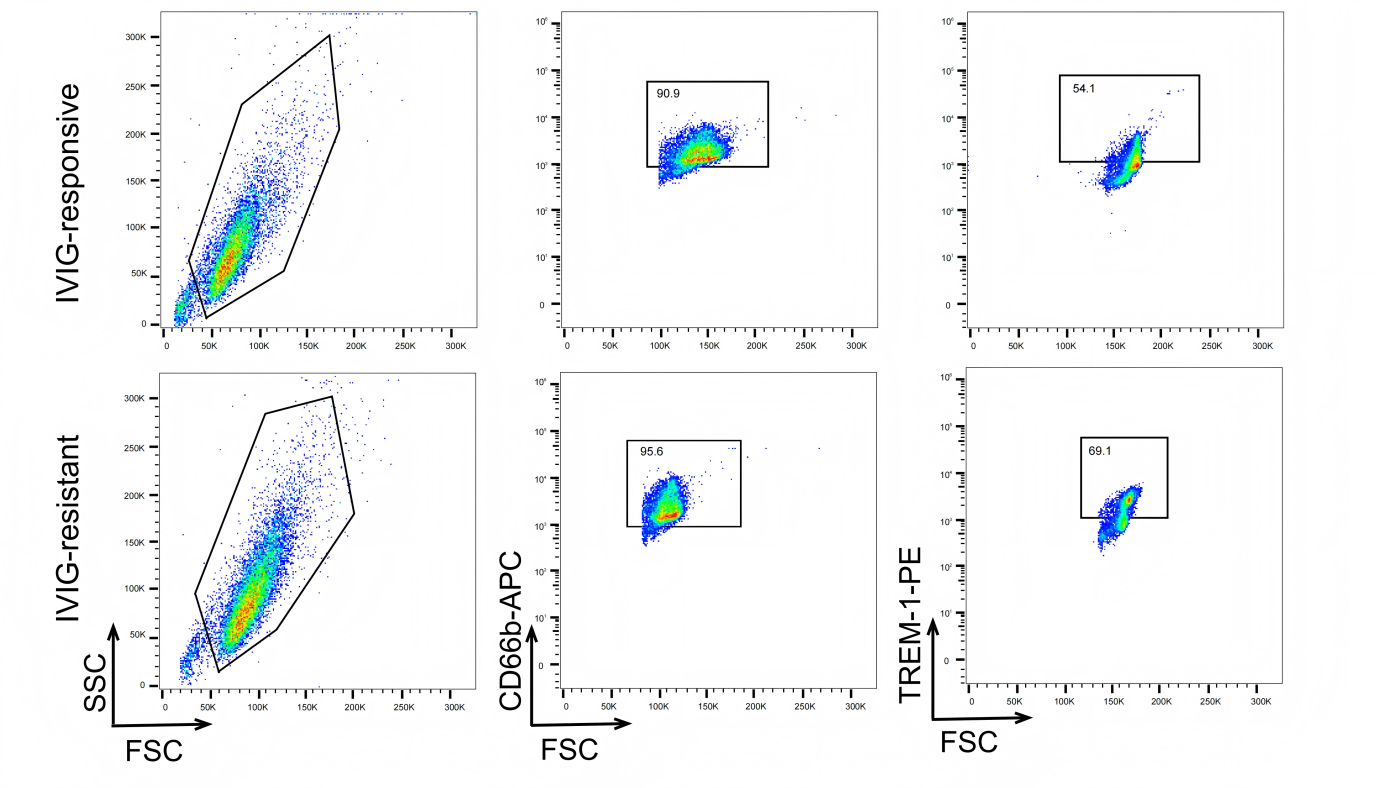


**Supplementary Figure S2.** Representative flow cytometry plots illustrating TREM-1 surface expression on neutrophils. Gating strategy: neutrophils identified by CD66b-APC positivity; TREM-1-PE expression shown within gated population.

DATA AVAILABILITY STATEMENT

**Transcriptomic Datasets:**

- GSE73461: Available at https://www.ncbi.nlm.nih.gov/geo/query/acc.cgi?acc=GSE73461
- GSE68004: Available at https://www.ncbi.nlm.nih.gov/geo/query/acc.cgi?acc=GSE68004
- GSE63881: Available at https://www.ncbi.nlm.nih.gov/geo/query/acc.cgi?acc=GSE63881

**Clinical Data:**
Clinical datasets generated in this study are available from the corresponding author upon reasonable request, subject to:

1. Institutional review board approval

2. Execution of appropriate data transfer agreements

3. Compliance with patient privacy protection regulations (GDPR, HIPAA equivalent)
